# V-DART: VR-Based Dual-Arm Robot Teleoperation with Mobile Base Control and Data Recording

**DOI:** 10.3390/s26196245

**Published:** 2026-10-01

**Authors:** Alejandro Torrejón, Jorge Calderón, Pedro Nuñez, Pablo Bustos

**Affiliations:** RoboLab, Robotics and Artificial Vision, University of Extremadura, 10003 Cáceres, Spain; atorrejon@unex.es (A.T.); calde@unex.es (J.C.); pbustos@unex.es (P.B.)

**Keywords:** virtual reality, teleoperation, dual-arm robotic manipulators, real-time visualization, immersive interfaces

## Abstract

We present V-DART (VR-based Dual-Arm Robot Teleoperation), an extended virtual reality framework for the teleoperation of mobile dual-arm robotic systems, building upon our previous work on stationary manipulator control. The enhanced system leverages Unreal Engine 5.6.1 (UE5) for real-time visualization and interaction, introducing critical advancements for complex environments: the control of the robot’s base movement via both joystick input and direct mapping of the user’s physical position and orientation within the tracked space. This allows users to navigate the robot intuitively by simply moving their body, while maintaining precise control over both arms using VR (Virtual Reality) controllers. Furthermore, we introduce a robust data logging layer that records expert movements during teleoperation tasks. By capturing timestamped streams of user pose, controller inputs, and robot states that can be synchronized downstream, this module facilitates the creation of high-quality datasets from expert demonstrations. These datasets can be leveraged for machine learning applications, such as behavior cloning, reducing the need for extensive manual annotation. The system maintains bidirectional communication between the VR interface and the physical robot, ensuring responsive control and real-time feedback. We describe the architectural enhancements, user interaction design, and performance evaluation, demonstrating the viability of this unified approach for both teleoperation and data-driven robotics development.

## 1. Introduction

### 1.1. Research Context

Teleoperation remains a cornerstone strategy for deploying robots in environments that are unstructured, hazardous, or otherwise unsuited to full autonomy, since it allows a human operator’s perception, judgment, and dexterity to be transferred to a remote or surrogate embodiment. Among the available interface modalities, Virtual Reality (VR) has emerged as a particularly compelling choice because immersive head-mounted displays and motion-tracked controllers provide depth perception, a first-person viewpoint, and direct spatial mapping between the operator’s body and the robot’s end-effectors, qualities that traditional 2D screen-and-joystick interfaces struggle to replicate [1,2]. As robotic platforms have grown more capable, however, the scope of teleoperation has expanded well beyond single, stationary manipulators toward mobile and bimanual systems, exposing new challenges in how an operator’s intent can be intuitively and reliably conveyed to a robot whose workspace is not fixed in space.

### 1.2. Recent Related Works

Early VR teleoperation systems largely targeted single-arm or collaborative manipulators performing pick-and-place or waypoint-based tasks [2,3]. Rosen and Jha showed that extending these practices to industrial arms is non-trivial, since proprietary position controllers and the absence of exposed torque or velocity interfaces require dedicated command-filtering strategies to achieve smooth, contact-rich manipulation [1]. Parallel efforts incorporated richer visual feedback, such as live point-cloud reconstructions or RGB-D (Red-Green-Blue-Depth)-based environment capture, to improve the operator’s situational awareness during remote manipulation [3,4]. More recent comparative studies have evaluated immersive VR against mixed-reality and conventional screen-based interfaces for legged and bimanual manipulators, generally reporting that VR improves operator confidence and decision-making at the cost of higher cognitive workload and longer task completion times in complex, multi-stage scenarios [5,6]. Building on this line of work, our own prior system introduced a unified VR architecture, using Webots simulation or a physical dual-arm robot within the same Unreal Engine 5.6.1 (UE5) application, that enabled an operator to control both manipulators’ tool points through motion-tracked controllers while preserving natural depth perception and real-time bidirectional synchronization between the virtual and physical domains [7].

Several recent platforms have addressed the coordination between mobile bases and manipulators. Mobile ALOHA augments a bimanual leader-follower teleoperation rig with a wheeled base actuated through the operator’s own body movement, recording synchronized base and arm trajectories to support whole-body imitation learning [8]. TeleMoMa explored a complementary set of input modalities, including VR controllers, vision-based body tracking, and space-mice, finding that hybrid schemes which let the operator choose among several control mappings improve both intuitiveness and precision [9]. More recent humanoid-oriented systems have gone further, streaming full-body pose estimates from VR headsets and ankle trackers directly into whole-body control policies, and explicitly comparing coupled versus decoupled base-arm embodiments, with evidence suggesting that coupled control, despite a comparable subjective workload, yields demonstration data that is more effective for downstream imitation learning [6,10].

A second driver reshaping the design of modern teleoperation systems is the growing reliance of robot learning on large volumes of high-quality human demonstrations. Behavior cloning, in which a policy is trained via supervised learning to reproduce the actions an expert took given the same observations, has become a standard and effective baseline for teaching manipulation skills, but its performance depends critically on the quantity and quality of the underlying demonstration data [11,12]. The Robomimic benchmark formalized this idea by curating large-scale, multi-operator demonstration datasets for standard manipulation tasks [12], while systems such as BEAVR have shown that VR teleoperation can be paired with zero-copy, low-latency streaming architectures to record synchronized multi-modal demonstrations directly in standardized dataset schemas, enabling compatibility with modern visuomotor policies [13]. For mobile bimanual platforms specifically, Mobile ALOHA demonstrated that simply concatenating base velocity commands with arm joint actions into a single action vector allows existing imitation-learning pipelines to be applied with minimal modification, achieving strong task performance after only a handful of expert demonstrations [8]. Beyond full end-to-end imitation learning, teleoperation-derived motion data has also been used to learn structured sub-components of a control pipeline; for instance, Shi et al. captured exoskeleton-based teleoperation demonstrations to train a lightweight network that predicts operator-specific redundancy parameters for a 7-DoF (Degrees of Freedom) humanoid arm, embedding this learned preference into a classical differential inverse-kinematics solver [14].

Finally, the choice of rendering and simulation backend has itself become an active design decision in teleoperation research. Game engines originally developed for interactive entertainment, particularly UE5, are increasingly adopted in robotics because their physically based rendering, GPU (Graphics Processing Unit)-accelerated lighting (Lumen), and geometry pipelines (Nanite) support the responsiveness required for human-in-the-loop control while also functioning as synthetic-data factories for downstream learning [15]. This has motivated several digital-twin teleoperation frameworks: IMMERTWIN couples UE5 with motion-planning back-ends to create a closed-loop digital twin in which discrepancies between commanded and actual robot states are continuously reconciled [16], while nuclear-inspection and humanoid digital-twin platforms have likewise relied on UE5 for real-time path visualization and high-fidelity scene replication [17,18]. Within this broader trend, our previous dual-arm system already established UE5 as a capable foundation for real-time, bidirectional VR teleoperation of a stationary manipulator pair [7].

Table 1 compares the hardware and interface configuration of V-DART against five related teleoperation systems for mobile and bimanual manipulation, covering robot mobility, teleoperation modality, visual feedback, and onboard sensing. Table 2 complements this hardware-level comparison by reporting, for the same systems, whether and how each explicitly supports logging demonstration data for downstream imitation learning, alongside the main experimental results reported in each study.

### 1.3. Limitations of Existing Methods

Despite these advances, existing systems still differ substantially in how locomotion, bimanual manipulation, visual feedback, and operator embodiment are combined. A persistent limitation of stationary-base systems, including our own prior work [7], is that they decouple the problem of arm control from the equally important problem of navigating the robot through its environment. Real-world tasks such as fetching an object from a shelf, opening successive doors, or repositioning before a manipulation sequence inherently require coordinated control of a mobile base alongside the arms, and treating these as independent control loops tends to produce stilted, sequential behavior rather than the fluid, whole-body motion a human performs naturally [8].

Conversely, mobile platforms that do achieve base-arm coordination, such as Mobile ALOHA [8] and TeleMoMa [9], typically rely on conventional or flat 2D video feedback rather than immersive 3D reconstruction, which limits the operator’s spatial perception of the remote environment. Immersive systems that do provide richer 3D visual feedback, such as IMMERTWIN [16] and our own prior dual-arm framework [7], have in turn predominantly focused on fixed-base robots. Humanoid-oriented systems that couple full-body embodiment with whole-body control [6,10] come closest to bridging this divide, but generally report increased task time and perceived workload, and do not incorporate a structured, multi-rate data-logging layer comparable to that required for downstream imitation learning. Overall, the human factors associated with combining mobile-base locomotion, bimanual manipulation, embodied VR interaction, and live 3D reconstruction, including task success, completion time, perceived presence, and cybersickness, remain less extensively characterized than in fixed-base VR teleoperation.

### 1.4. Research Gap

Consequently, relatively little work has explored a unified interface that combines mobile-base locomotion, bimanual manipulation, embodied VR interaction, and live 3D reconstruction within a single teleoperation system, together with a logging layer capable of directly producing machine-learning-ready demonstration data.

### 1.5. Objective of This Study

These observations motivate V-DART, which extends immersive VR teleoperation toward a unified mobile dual-arm interface while also providing live 3D reconstruction of the remote environment. The objective of this study is to design, implement, and experimentally evaluate such an interface, assessing its viability both for real-time teleoperation and for the generation of expert demonstration datasets.

### 1.6. Novelty

Building on this foundation, the present work introduces V-DART (VR-based Dual-Arm Robot Teleoperation), which extends our prior stationary dual-arm framework [7] to mobile dual-arm robotic systems. Unlike prior mobile teleoperation systems, which largely rely on conventional or flat video feedback, and unlike prior immersive VR systems, which have remained confined to fixed-base manipulators, V-DART is, to our knowledge, the first system to jointly combine (i) coordinated mobile-base and bimanual VR teleoperation, (ii) live 3D (LiDAR + RGB-D) reconstruction of the remote environment within the VR headset, and (iii) a structured, timestamped, multi-rate data-logging layer designed to be directly compatible with behavior-cloning and related imitation-learning pipelines, all within a single UE5-based architecture.

### 1.7. Main Contributions

The contribution of this work is threefold. First, we generalize the control architecture to support robot base locomotion through two complementary modalities, joystick input and direct mapping of the operator’s physical position and orientation within the tracked space, allowing the user to navigate the robot simply by walking while retaining precise, independent control of both arms via VR motion controllers. Second, we introduce a data-logging layer that records timestamped streams of user pose, controller inputs, and robot state throughout each teleoperation session, enabling the reconstruction of synchronized tuples for downstream use and transforming every expert demonstration into a structured, machine-learning-ready dataset designed to be compatible with behavior cloning and related approaches, without requiring separate manual annotation. Training or evaluating a policy on this data is not performed in this work and is identified as a direction for future work in Section 4.7. Third, we preserve and extend the bidirectional, real-time communication between the VR interface and the physical robot established in our earlier work, ensuring that the added mobility and logging capabilities do not compromise the responsiveness of the original system. The remainder of this paper details the architectural enhancements that enable this unified mobile dual-arm interface, the design of the user interaction and data-logging layers, and an experimental evaluation that assesses the viability of the approach for both real-time teleoperation and the generation of expert demonstration datasets.

## 2. Materials and Methods

This section describes the experimental platform on which V-DART (version 1.0.0) is deployed and evaluated. We first introduce P3Bot, the mobile dual-arm robot used as the physical target of the teleoperation system (Section 2.1), and the operator workstation that hosts the VR application (Section 2.2). We then detail the internal architecture of V-DART, including its entity-component organization within Unreal Engine 5.6.1 and the middleware abstraction layer that decouples the immersive interface from the underlying robotics communication stack (Section 2.3). Although the present work focuses on P3Bot, the same architecture has previously been used to teleoperate a stationary Kinova-based setup [7], illustrating the multi-robot scalability of the framework. Finally, we describe the binary data-recording layer used to capture expert demonstrations for downstream learning applications.

### 2.1. P3Bot

The robotic platform used in this study is P3Bot [19], a mobile dual-arm robot developed in-house by the RoboLab research group at the University of Extremadura, Spain. P3Bot shares its power electronics, omnidirectional base, and part of its perception system with Shadow [20], an earlier social robot developed by the same group.

**Figure 1:**
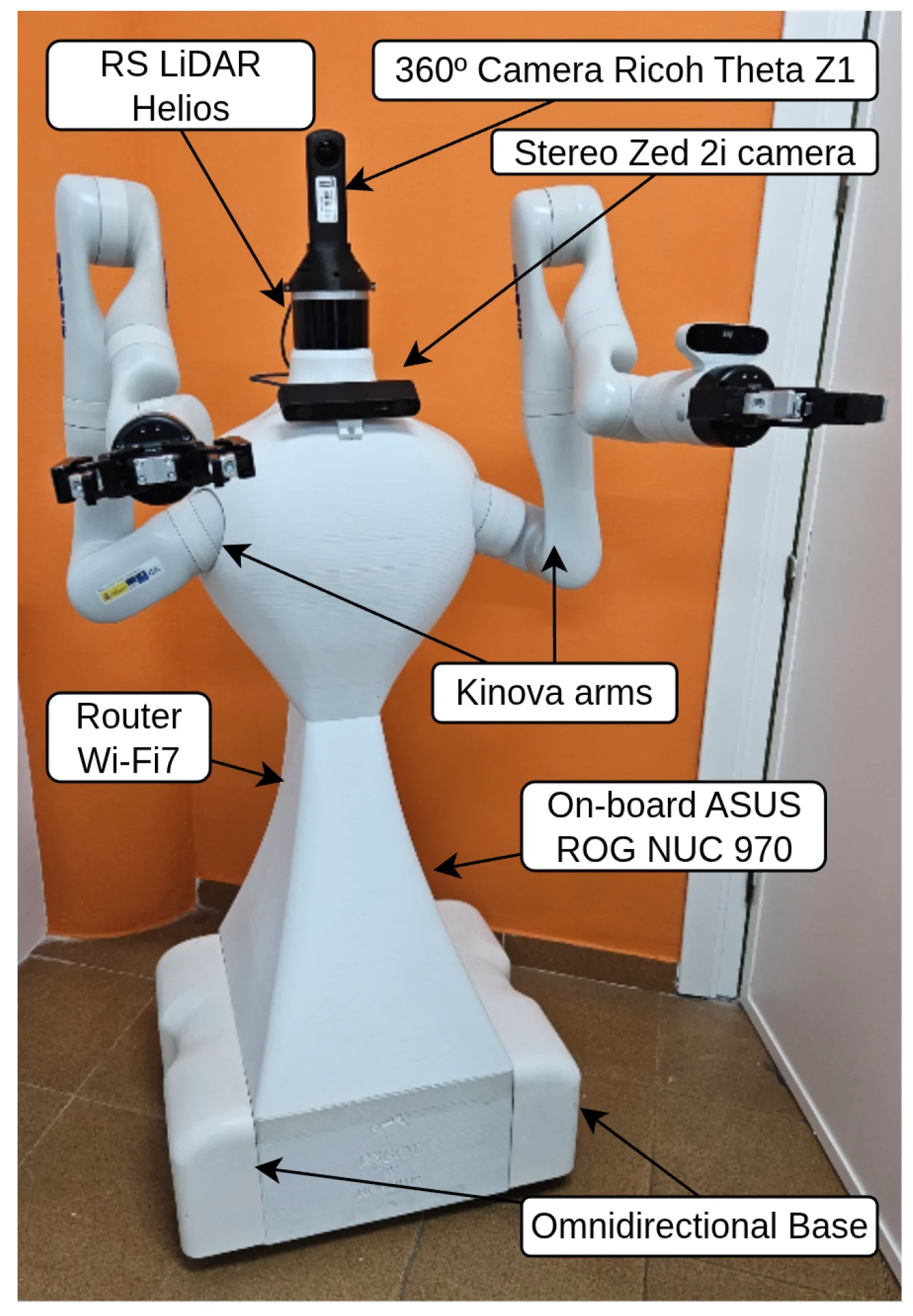
Hardware components of the P3Bot mobile dual-arm robot.

P3Bot, shown in Figure 1, is built from three PLA parts produced by additive manufacturing (FDM 3D printing) and integrates the hardware components mentioned Table 3.

For safety reasons, the platform’s motion is constrained by software velocity limits. The mobile base’s linear velocity is limited to 0.8 m/s and its angular velocity to 1.5 rad/s, while the arm joint velocities are limited to 50°/s.

### 2.2. Operator Workstation and Communication Topology

V-DART runs on a dedicated workstation, physically independent of P3Bot’s on-board computer, equipped with an Intel Core i9-14900KF CPU (Intel Corporation, Santa Clara, CA, USA) (Central Processing Unit) and an NVIDIA RTX 4080 GPU (NVIDIA Corporation, Santa Clara, CA, USA). This workstation is responsible for executing the Unreal Engine 5.6.1 application, driving the immersive interface, and exchanging data with the robot in real time. The VR head-mounted display is a Meta Quest 3, connected to the workstation through ALVR over a USB cable, which streams rendered frames to the headset at 2272×2352 pixels per eye at a refresh rate of 90 Hz.

**Figure 2:**
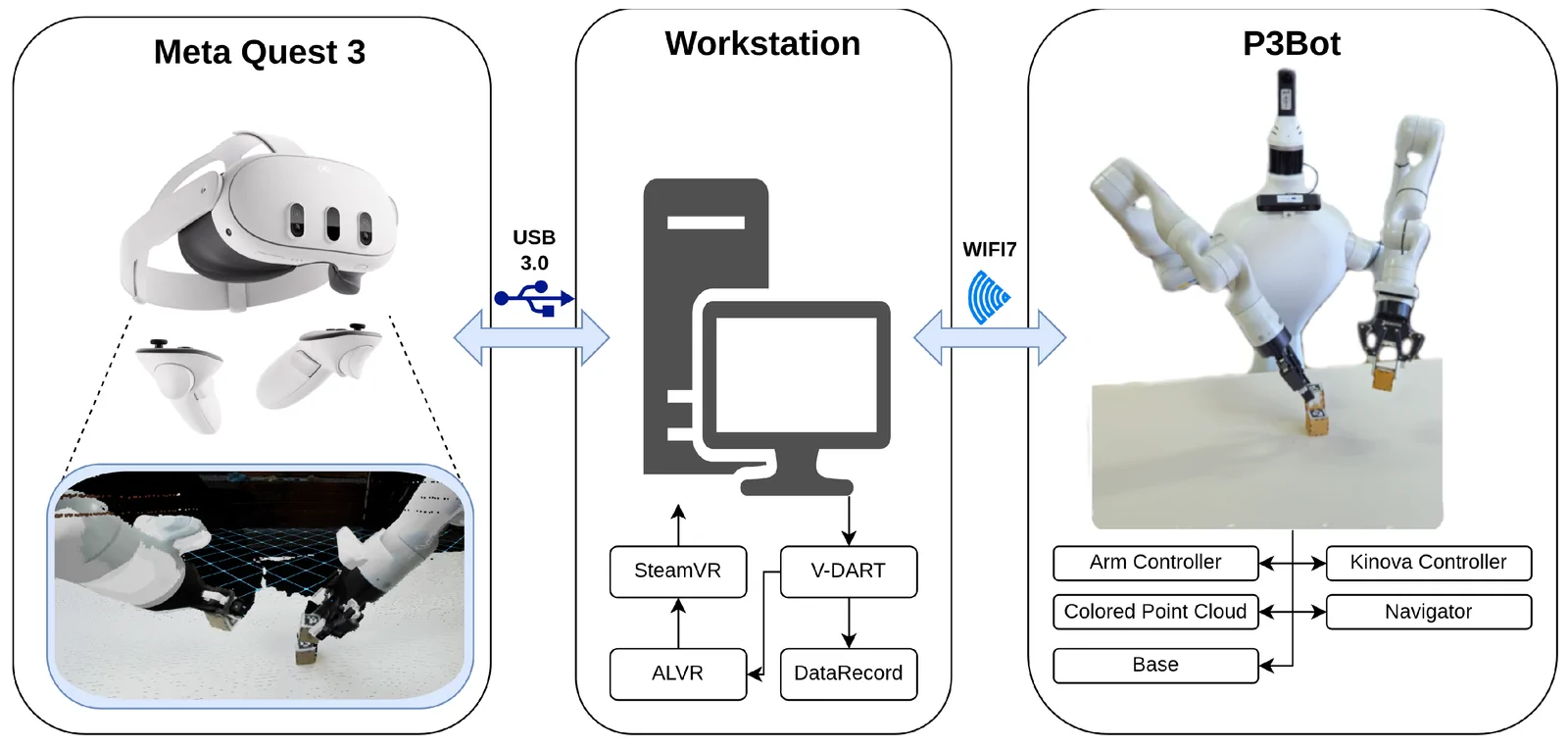
Communication topology between the Meta Quest 3 headset, the V-DART workstation, and the P3Bot robot.

The complete communication topology between the headset, the workstation, and the robot is depicted in Figure 2. Table 4 summarizes the data streams transmitted by P3Bot to the workstation over Wi-Fi 7.

In the opposite direction, the workstation transmits the control commands summarized in Table 5 to the robot.

**Figure 3:**
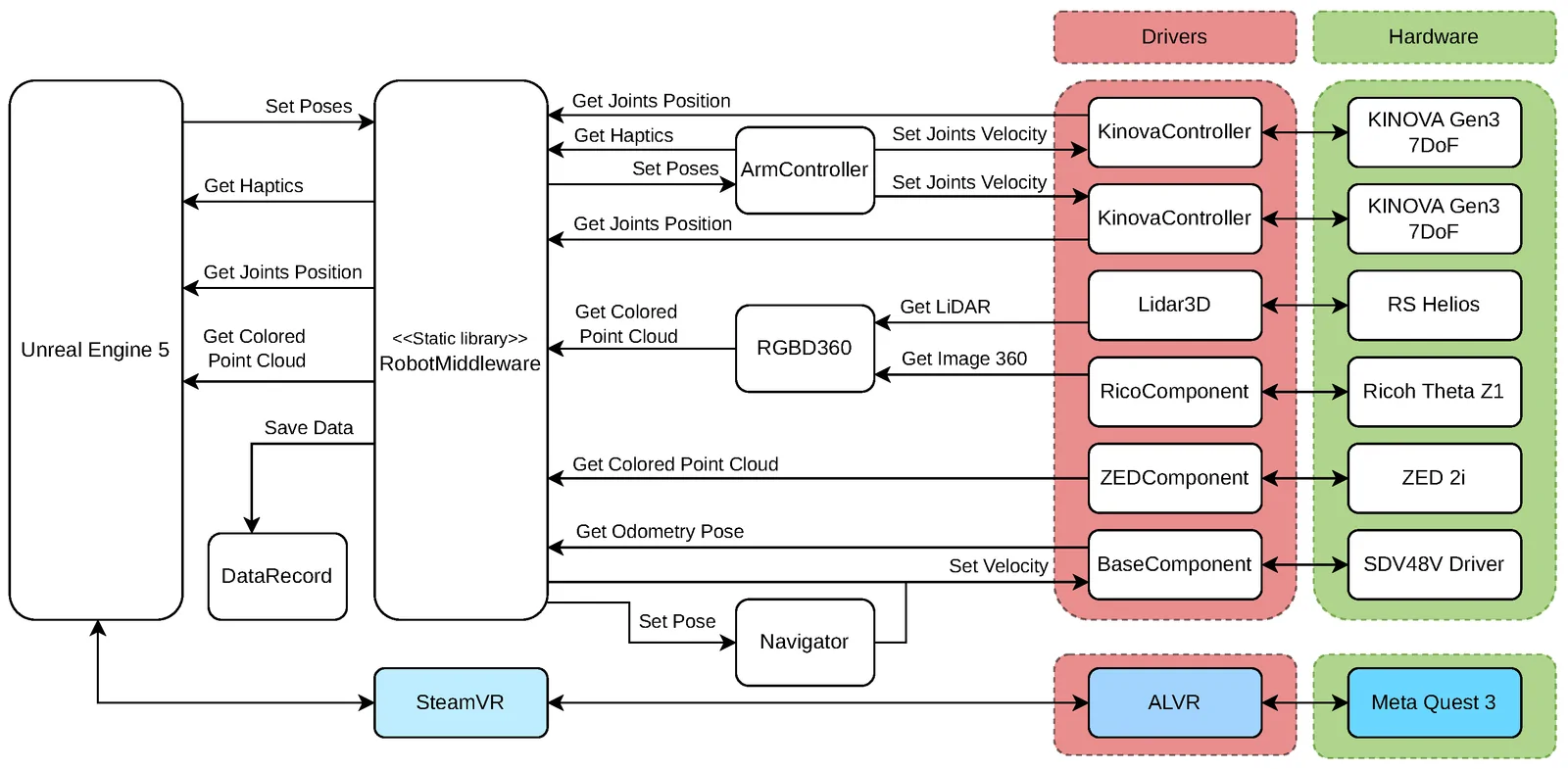
Communication and component overview of the proposed system. UE5 interacts with the robotics back-end only through RobotMiddleware (static library), which bridges the XR application with RoboComp/Ice modules. Driver components (red) interface the physical devices (green): two Kinova Gen3 arms (via KinovaController), RS Helios LiDAR (via Lidar3D), Ricoh Theta camera (via RicoComponent), ZED 2i stereo camera (via ZEDComponent) and robot base (via BaseComponent). The Meta Quest 3 headset is connected through ALVR and exposed to UE5 via SteamVR. Adapted from [7] (CC BY 4.0).

Using these data streams, the workstation reconstructs the perceived world inside UE5, augments it with the virtual representation of the robot, and streams the resulting stereoscopic rendering to the Meta Quest 3. The software components involved in this pipeline, and their interfaces with the physical devices, are shown in Figure 3.

### 2.3. V-DART Architecture

**Figure 4:**
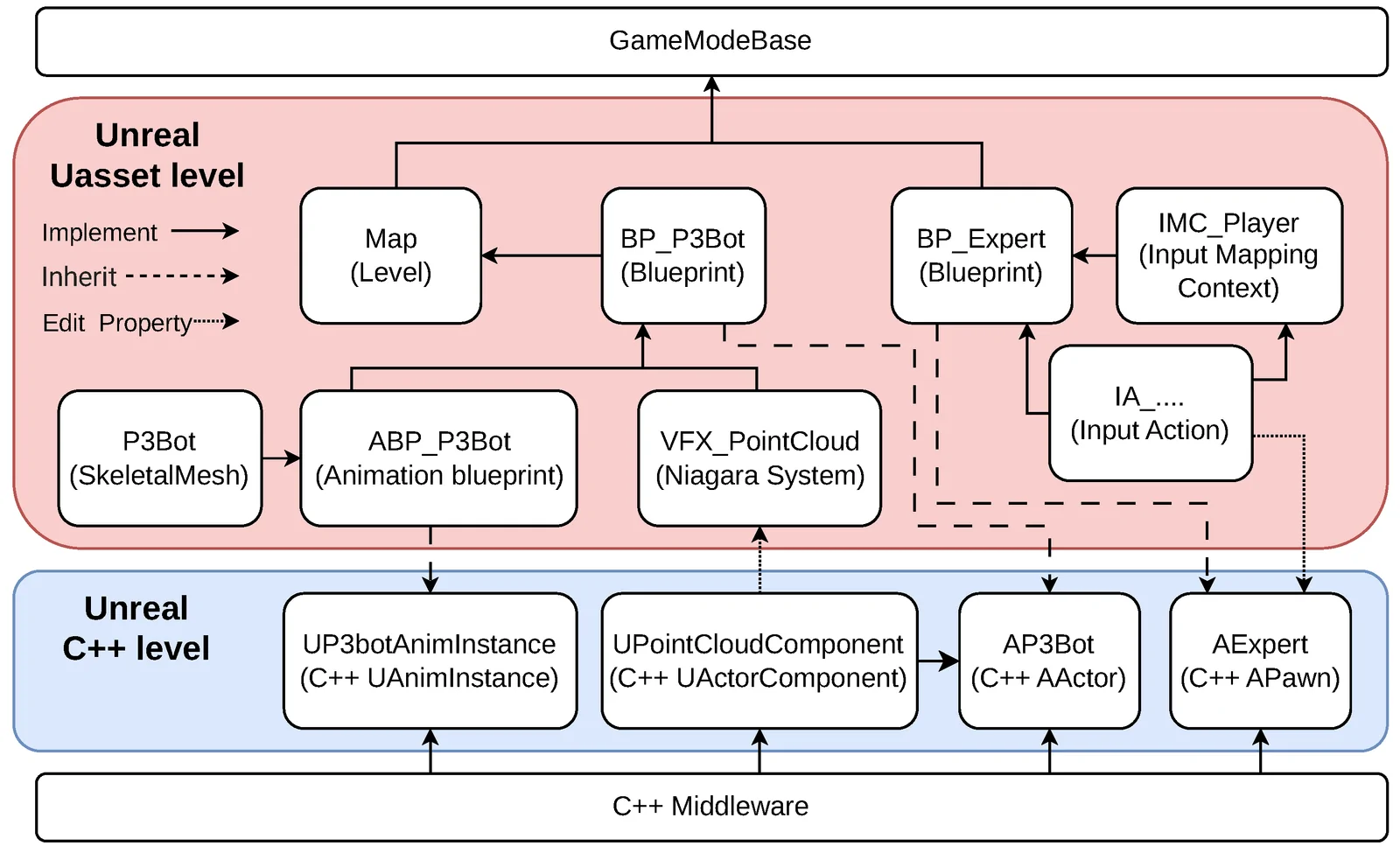
Schema of entities and components composing the V-DART architecture.

As previously stated, V-DART (the V-DART source code is available at https://github.com/alfiTH/V-DART (accessed on 21 September 2026)) is built on top of Unreal Engine 5.6.1, compiled with Clang 18.1.3 and the -std=c++latest standard flag. UE5 was selected over alternative game engines because of its superior rendering performance and its suitability for real-time, human-in-the-loop robotics applications, as reported in recent comparative analyses [21,22]. The internal organization of V-DART, summarized in Figure 4, follows the Entity-Component paradigm native to UE5.

#### 2.3.1. V-DART Entities and Components

The internal architecture of V-DART is composed of the following entities and components:

- **SkeletalMesh**: defines the kinematic structure and visual mesh of the robot inside UE5.
- **Animation Blueprint**: a C++-based UAnimInstance that binds the incoming joint angles of both arms to the corresponding joints of the SkeletalMesh, ensuring that the virtual robot mirrors the physical configuration in real time.
- **Niagara System**: a GPU-accelerated rendering subsystem, implemented as a C++ UActorComponent, that efficiently displays the colored point clouds produced by the on-board sensors. Particles are instantiated once and updated through vectorized operations, enabling interactive frame rates even with multi-million-point clouds.
- **Level**: the Unreal world in which the robot is placed, including lighting, environmental geometry, and the operator’s VR pawn.
- **Input Mapping Context**: aggregates the Input Actions associated with the VR controllers and headset, mapping them to high-level operator commands.
- **Expert (Player) Blueprint**: a C++-based APawn representing the operator inside the virtual world. It hosts the input handling logic and the components responsible for tracking the operator’s pose.
- **Robot Blueprint**: a C++-based AActor that encapsulates the SkeletalMesh, the Animation Blueprint, and the Niagara System described above, exposing them as a single coherent representation of the physical robot.
- **GameModeBase**: the orchestrating entity that initializes the world, instantiates the expert pawn and the robot actor, and coordinates the overall execution loop.

#### 2.3.2. Controller Input Mapping

**Figure 5:**
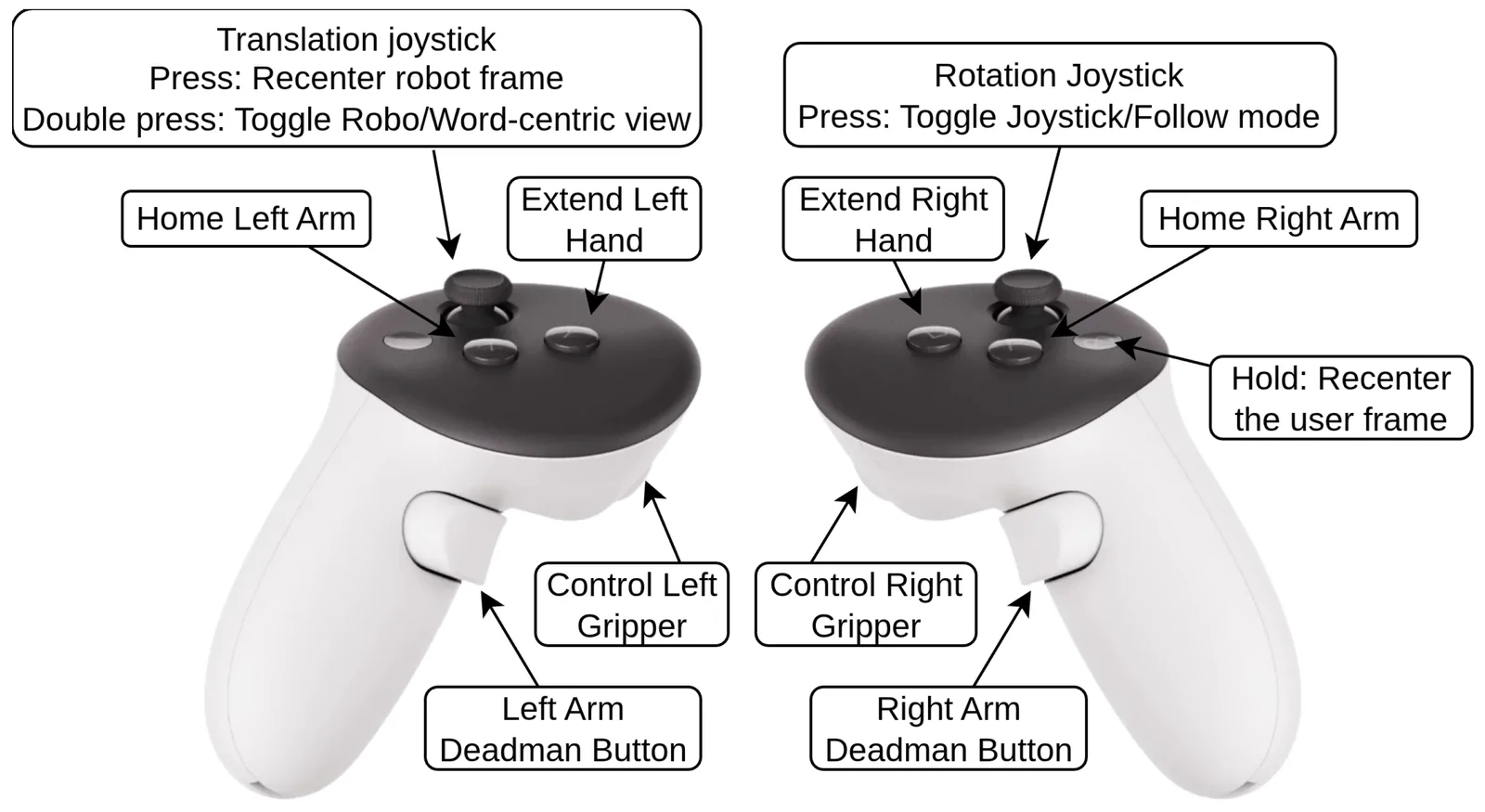
V-DART input mapping on the Meta Quest 3 controllers. Each hand drives one arm through the corresponding deadman button and trigger, while the joysticks are dedicated to base locomotion and view management.

The input mapping used by V-DART is summarized in Figure 5. Bimanual manipulation is performed by moving each controller through space: the pose of each controller in the tracked area is mapped to the tool-point set-point of the corresponding Kinova Gen3 arm, provided the associated deadman button is being held down. This deadman scheme prevents unintended motion when the operator releases the controllers or takes them off, following standard safety practice in VR-based robot teleoperation [7,16]. The conversion from these Cartesian tool-point set-points to joint-space commands is performed by a numerical inverse-kinematics solver built on Peter Corke’s Robotics Toolbox [23], which computes smooth joint trajectories across the arm’s workspace with virtually no singular configurations. Gripper opening and closing is bound to the trigger of each controller, so that each hand fully drives one arm end-effector without cross-coupling. Two auxiliary buttons per controller (home and extend) modulate the arm-control mapping. The home button returns the corresponding arm to a canonical resting pose, providing quick recovery from awkward joint configurations. The extend button virtually extends the reach of the operator’s hand within UE5 along the direction the controller is pointing, so that far tool-point targets remain reachable without the operator physically walking towards them. This design decision addresses the mismatch between the human arm span (approximately 0.5 m of comfortable reach in each direction from the shoulder) and the P3Bot arm workspace (approximately 1 m radius from each shoulder joint): without this mechanism, the operator would need to displace their entire body to command tool-point set-points near the outer limits of the robot’s workspace, which is impractical inside a bounded VR play area and disrupts the mapping between operator pose and base-follow mode.

Base motion is mapped to the joysticks of the two controllers: the left joystick commands planar translation of the mobile base, while the right joystick commands its rotation around the vertical axis. Pressing the translation joystick recenters the robot frame, so that subsequent joystick motions are interpreted relative to the operator’s current orientation, and a double press of the same joystick toggles between the two viewpoint modes described in Section 2.3.3. Pressing the rotation joystick toggles between direct joystick control of the base and a follow mode in which the base autonomously tracks the operator’s own displacement within the tracked area. Finally, holding a dedicated button on the right controller recenters the operator’s own frame of reference within the virtual world, which is useful when the physical play area and the virtual environment become misaligned during long sessions.

#### 2.3.3. Viewpoint Modes

V-DART supports two complementary viewpoint modes, toggled at runtime through a double press of the translation joystick.

##### World-Centric View

The virtual robot is rendered at its true pose within the reconstructed environment and translates through the virtual world as the physical robot navigates. The operator observes the scene from a static vantage point and can invoke a recenter action to re-anchor the view to the robot’s frame whenever it moves out of comfortable viewing range. This mode decouples the operator’s vestibular system from the robot’s motion and is therefore recommended for users prone to motion sickness or new to VR.

##### Robot-Centric View

The virtual robot remains anchored at the world origin and the reconstructed environment moves around it in the opposite direction as the physical robot navigates. This mode maximizes the sense of embodiment, since the operator’s body and the robot’s chassis share a common frame of reference at all times. However, the induced optical flow without a corresponding vestibular signal is a well-known cause of vection-induced cybersickness in immersive VR [24], which we observed in less experienced operators during long-range navigation trials.

Both modes share the same input mapping, the same manipulation semantics, and the same data-logging pipeline, so that demonstrations recorded in either mode remain directly comparable.

#### 2.3.4. Communication Middleware

To decouple the immersive layer from the underlying robotics communication stack, the network and control logic is encapsulated in a shared library named RobotMiddleware.so. This library exposes a stable C++ interface to UE5, providing both the robot state required for rendering and the control commands generated from the operator’s input. The current implementation relies on RoboComp [25], a component-oriented framework built on top of the ZeroC ICE (Internet Communications Engine) middleware, but the abstraction is general and can be re-targeted to alternative middleware such as ROS (Robot Operating System) or ROS 2 without modifying the UE5 application.

#### 2.3.5. Expert Demonstration Recording

V-DART includes a dedicated data-logging layer that captures expert demonstrations during teleoperation sessions. Rather than emitting one synchronized tuple per control tick, the logging layer records a stream of independent, timestamped records: each time RobotMiddleware receives or generates a relevant data—whether an input sample from one of its own polling loops (VR head/controller pose, arm joint state, robot pose received from P3Bot) or an output command issued from Unreal’s tick (commanded tool-point set-points, base motion commands)—an independent record is emitted. The synchronized tuple for a given instant is reconstructed downstream by grouping records from the different channels that share the same timestamp, rather than being captured as a single atomic record per cycle.

**Figure 6:**
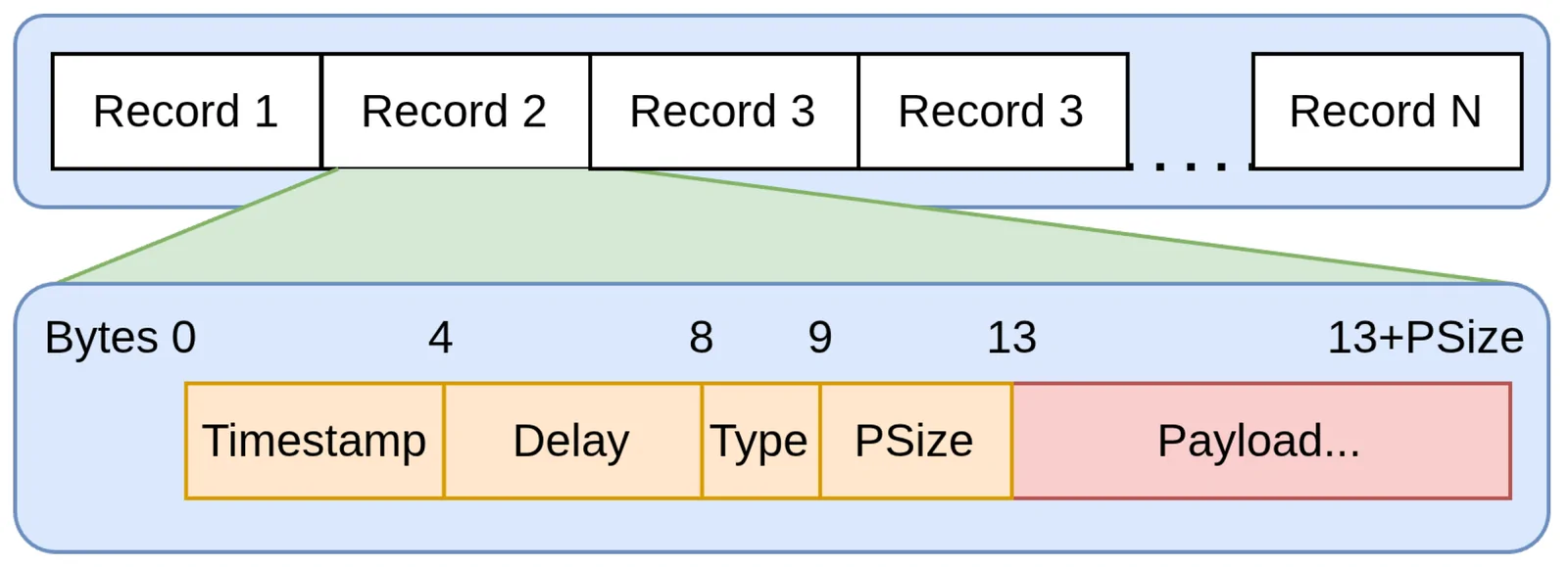
Structure of the concatenated record stream (**top**) and byte-level layout of the 13-byte RecordHeader (**bottom**). The header is shown in yellow and the payload in red.

Rather than encoding demonstrations with a schema-based serializer (JSON, Protocol Buffers, etc.), the data-logging layer writes the in-memory layout of each C++ record struct directly to disk via a raw memcpy. The resulting file is a flat, self-describing sequence of Type-Length-Value records concatenated with no inter-record separator and no file-level header or index, as shown in Figure 6. Each record consists of a RecordHeader (yellow), whose fixed 13-byte layout is detailed in Table 6, followed by a payload (red), which is the packed binary image of one of the structs listed in Table 7. There is no global index or total file length: a reader must scan the stream sequentially, parsing each header to determine how many payload bytes follow.

##### RecordHeader

The header is fixed at 13 bytes, with byte-level packing enforced via #pragma pack(push,1) to eliminate compiler-inserted alignment padding. The field layout is shown in Figure 6 and summarized in Table 6.

##### Record Types and Payload Sizes

For a given RecordType, the payload size is fixed at compile time (with the exception of ColorCloudData), which allows each record to be parsed in O(1) without tokenizing text.

The base structs composing these payloads are: Pose (28 B: seven floats – translation (x,y,z) and orientation quaternion $\left(q_{x},q_{y},q_{z},q_{w}\right)$); Controller (31 B: seven floats for trigger/grip/thumbstick axes and capacitive touch, plus three bools for button state); Haptic (8 B: intensity and frequency); and ArmJoint (32 B: a float[8] holding seven joint angles plus gripper aperture).

##### Design Properties

- **Heterogeneous trigger sources.** Not all records are emitted at the same cadence or by the same actor: output records (BaseSpeedCmd, BasePoseCmd, commanded tool-point set-points) are written synchronously with Unreal’s own tick, since they originate in the game engine’s command-issuing code, whereas input records (VRPose, VRController, VRHaptic, ArmJoints, RobotPose) are written from RobotMiddleware’s independent polling/read loops, each potentially running at its own rate. The shared timestamp field is therefore the only synchronization primitive across an otherwise asynchronous, multi-rate set of sources.
- **No padding.** Every struct in the hierarchy is packed with #pragma pack(1), so sizeof(T) equals the exact sum of its fields, with no compiler-inserted alignment bytes.
- **Zero-cost encoding.** Because serialization is a direct memcpy of the in-memory layout (no field-by-field marshaling), the cost of logging a sample is O(sizeof(T)) with no parsing or heap allocation, allowing recording at control-loop frequency without impacting VR responsiveness.
- **Not architecture-portable.** Since neither byte order nor ABI (Application Binary Interface) layout is fixed explicitly, a recorded file is only guaranteed to be valid when re-read on the same architecture (x86-64, little-endian) and compiler/flags that produced it; this is an internal log format rather than an interchange format such as Protocol Buffers or FlatBuffers.
- **Self-describing records, no global index.** Each record knows its own length (payloadSize), but the file carries no table of contents or total length, so random access to the *k*-th record requires a sequential scan from the start.
- **Synchronized tuples via timestamp join, not atomic records.** Each call to addData writes one independent record regardless of its trigger source; the synchronized tuple of VR pose, input actions, commanded set-points, and robot state is reconstructed downstream by grouping records that share the same timestamp (all referenced to the single elapsedMs() clock started by startRecording()).

#### 2.3.6. Multi-Robot Support

Although the present study focuses on the mobile dual-arm P3Bot platform, V-DART is not tied to any specific embodiment. Adding a new robot requires only three elements: (i) a SkeletalMesh describing its kinematic structure, (ii) an Animation Blueprint that binds the incoming joint states to the corresponding joints of the mesh, and (iii) a middleware endpoint exposing the robot’s state and accepting the control commands defined in Section 2.3.4. Under this abstraction, we have previously teleoperated a stationary single-arm Kinova Gen3 mounted on a workbench (video demonstration available at https://youtu.be/dXHj8Pkrrvo (accessed on 21 September 2026)) using the same UE5 architecture [7], differing from the P3Bot configuration only in the disabled base-motion channel and the reduced number of active arms. The same procedure applies to bimanual stationary setups, mobile single-arm platforms, or robots based on manipulators other than the Kinova Gen3 family, provided the corresponding middleware bindings are supplied through the shared library described in Section 2.3.4. This makes V-DART a general-purpose immersive teleoperation front-end rather than a P3Bot-specific tool.

### 2.4. Time Alignment and Latency Handling

Keeping the visual stream and the physical motion of the platform in step is essential for a coherent teleoperation experience. To this end, every machine on the local network is time-aligned through the Precision Time Protocol (PTP), reaching sub-millisecond clock offsets across devices. Building on this shared time base, the RobotMiddleware relies on a timestamped buffering mechanism that pairs each incoming point-cloud frame from the ZED 2i camera with the corresponding kinematic state of the robot at the moment of capture.

The ZED 2i camera remains the main source of dense spatial information for the operator, but it also imposes a measurable delay: the end-to-end latency, from sensor capture to final rendering inside the VR headset, was measured at approximately 143 ms. Although this delay is perceptible, the overall pipeline still delivers a stable and consistent visual experience, since the sub-millisecond PTP alignment guarantees that the spatial data reaching the headset is always internally coherent. In addition, the rendering of the robot’s virtual mesh is handled independently from the point-cloud pipeline: whereas the point-cloud reconstruction inherits the full sensing latency, the mesh representation of the robot is refreshed almost instantly based on direct joint-encoder and odometry data. This decoupling gives the operator a near-zero-latency visual reference for the arms, which helps offset the perceptible lag of the point-cloud stream.

A different behavior, however, appears in the mobile base. Unlike the manipulators, whose virtual mesh is driven directly by joint-encoder data, the base’s odometry is published at 100 Hz, while the camera feed used to reconstruct the point cloud operates at only 30 Hz. This frequency mismatch becomes noticeable specifically during rotational movements: since the base’s pose is updated faster than the corresponding point-cloud frame, the point cloud momentarily lags behind the actual position of the base, producing a transient spatial lag between the visual representation and the true robot pose. Notably, this lag is imperceptible in the Robot-centric view (defined in Section 2.3.3), since in this view the robot is kept fixed at the world origin, and it is the surrounding environment that moves relative to it rather than the robot moving within the scene.

### 2.5. Experimental Design

To evaluate V-DART, four demonstration experiments were designed, and eight participants took part in this experiment, with ages ranging from 25 to 34, of whom 6 had no prior experience with VR and 2 had extensive VR experience, each stressing a different combination of the control and visualization modes described in Section 2.3.2 and Section 2.3.3. The tasks are detailed below (a video recording of the four experiments is available at https://youtu.be/u4qYwOY5sj0 (accessed on 21 September 2026)).

**Figure 7:**
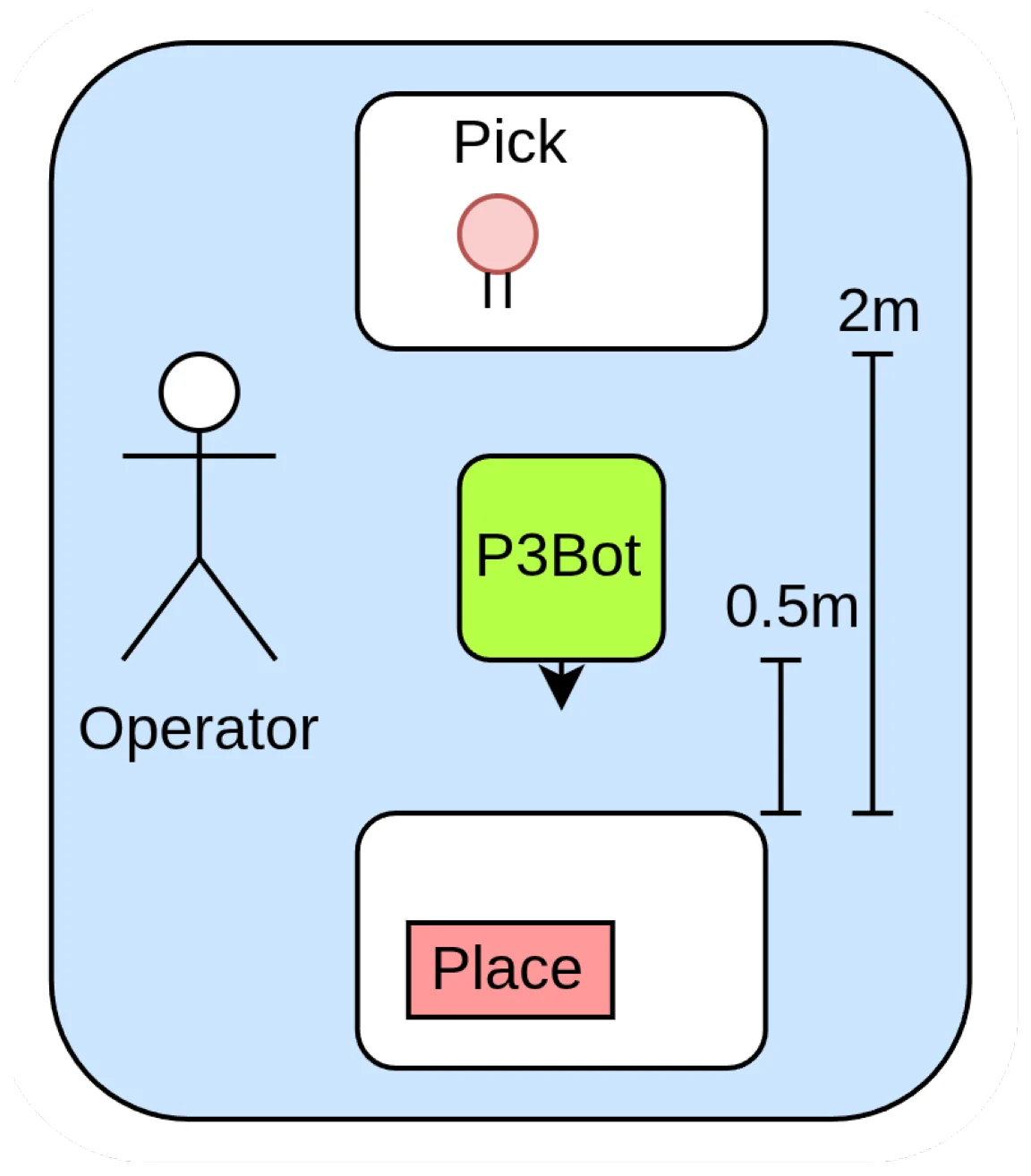
Physical layout of Experiment 1: short-range pick-and-place with operator and robot co-located in the same room.

**Figure 8:**
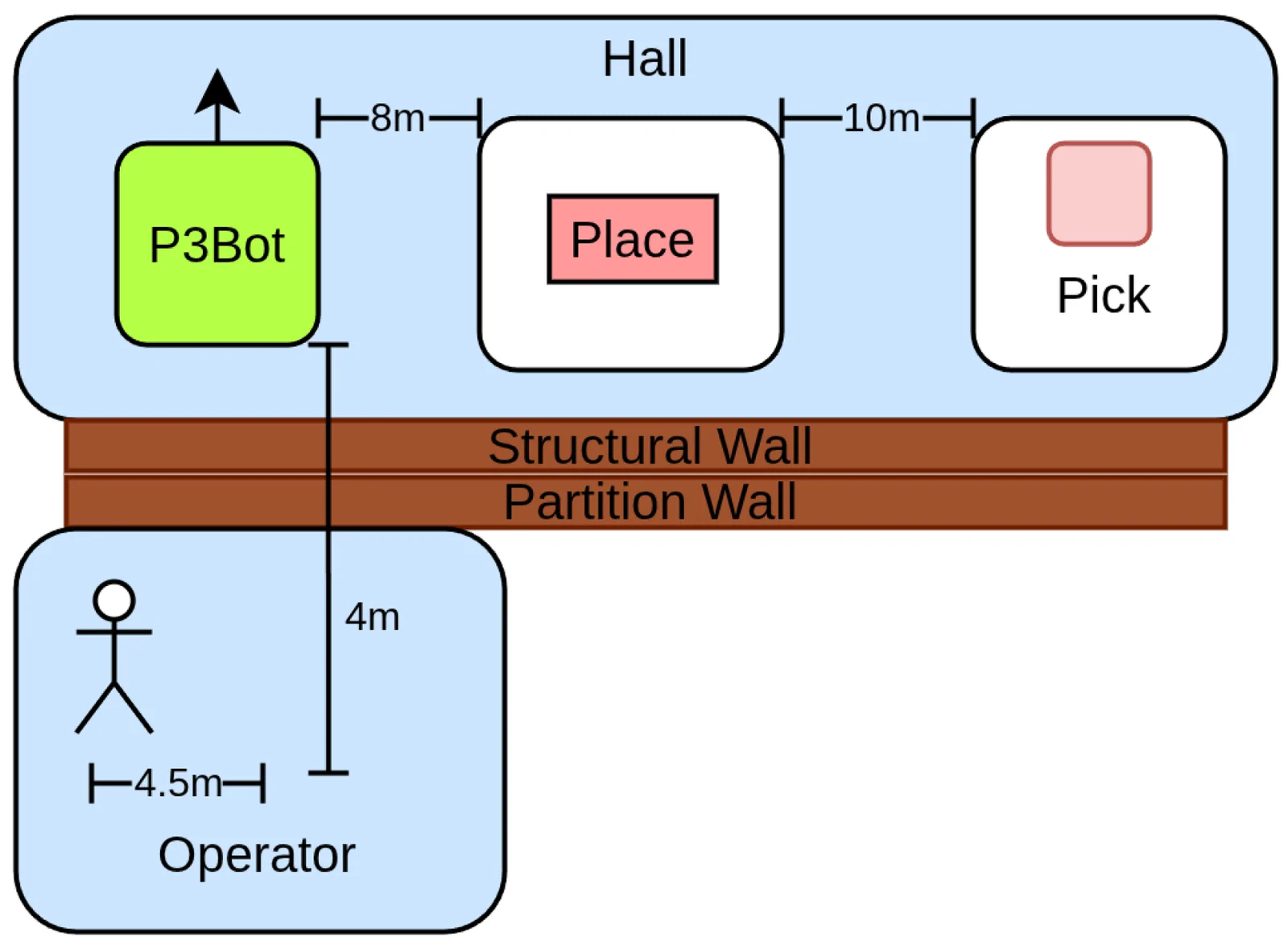
Physical layout of Experiments 3 and 4: long-range mobile manipulation along a hall, with the operator in a separate room connected through a doorway.

- **Experiment 1—Short-Range Pick-and-Place.** The robot must pick a ping-pong paddle from one table and deposit it inside a box placed on a second table located 2 m away from the first. See Figure 7 for the physical layout. Base motion is driven by the operator’s own displacement within the tracked area (follow mode), while both arms are controlled through the VR controllers. The visualization uses the robot-centric view. This experiment targets short-range, dexterous bimanual manipulation with minimal locomotion, and additionally stresses the vertical precision required by the constrained deposit target (the box opening).
- **Experiment 2—Passing through doors.** In this experiment, the robot starts positioned in front of a doorway with a width of 80 cm, while the P3Bot has a width of 74 cm, leaving a clearance of only 6 cm (approximately 3 cm on each side). The robot must traverse this first doorway and, subsequently, a second one under the same conditions. The goal of this experiment is to validate the precision and the quality of the visualization and control of the robot’s base when operating in narrow passages with minimal lateral clearance.
- **Experiment 3—Long Range Mobile Manipulation, World-Centric View.** The robot starts in the hall and must traverse it to reach the pick station located 18 m from its starting pose, pick up a bag placed on it, and return to the place station located 8 m from the origin (i.e., 10 m back from the pick station). The operator is located in a separate room connected to the hall through a doorway, without direct line of sight to the robot. The straight-line distance between the operator and the robot varies from approximately 6.0 m at the starting pose to 22.9 m at the pick station, with the wireless link crossing one load-bearing wall and one partition wall throughout the trial. See Figure 8 for the physical layout. Base motion is driven by the joysticks and manipulation by the VR controllers. The visualization uses the world-centric view with periodic recentering actions.
- **Experiment 4—Long Range Mobile Manipulation, Robot-Centric View.** Identical to Experiment 3 in task, environment, and control modality, but performed under the robot-centric visualization. The comparison between Experiments 3 and 4 isolates the effect of the visualization mode on task performance and operator comfort while keeping the task and control scheme constant. Since joystick operation does not involve the VR headset, the joystick condition is invariant to the choice of visualization mode; Experiment 4 therefore reuses the joystick trial from Experiment 3 as its baseline and reports data only for the VR (robot-centric) condition.

Physical setup. Experiments 2, 3 and 4 are conducted with the operator in a separate room from the robot, connected to the hall where the robot operates through two doorways, without direct line of sight and crossing one load-bearing wall and one partition wall. In these three experiments, the straight-line operator-to-robot distance varies from approximately 6.0 m at the starting pose to 22.9 m at the farthest reach of the trajectory. Experiment 1 is instead co-located: the operator and the robot share the same room. All experiments communicate exclusively through the Wi-Fi7 link described in Section 2.2, so that the results characterize V-DART as a genuinely remote teleoperation system rather than a co-located VR interface. Room dimensions, table placement, and target-object positioning were kept constant across trials within each experiment.

Each task was segmented into four consecutive phases for timing analysis:

- **Go to pick**: from the start of the trial until the robot’s base reaches a position from which the arm can engage the object.
- **Pick**: from the onset of arm motion until the object is fully lifted off the table.
- **Go to place**: from the end of the pick phase until the robot’s base arrives within reach of the place location.
- **Place**: from the onset of arm motion toward the target location until the object is released at the designated position.

All experiments were evaluated under two different control conditions. The first condition used two Xbox controllers: one controller was used to control the robot’s base motion, while the other was used to control one of the two robotic arms (hereafter, the joystick condition). The second condition, which constitutes the primary focus of this study, used a VR headset and its associated controllers for robot teleoperation (hereafter, the VR condition).

Participants were given seven minutes to familiarize themselves with each control scheme (joystick and VR controllers) prior to data collection.

### 2.6. Statistical Analysis

Given the small paired sample (n=8 participants completing both conditions within each experiment), statistical comparisons between conditions were performed using the paired Wilcoxon signed-rank test. Reported *p*-values are uncorrected for multiple comparisons, given the exploratory nature of this initial evaluation. Consequently, all *p*-values reported throughout this manuscript should be interpreted as descriptive, hypothesis-generating evidence rather than confirmatory statistical significance, and results are explicitly labeled as significant only when *p* falls below the nominal 0.05 threshold without adjustment.

## 3. Results

### 3.1. Performance Results

Before evaluating V-DART on teleoperation tasks, we characterized the rendering pipeline under sustained operation with the full sensor payload of P3Bot active. On the workstation described in Section 2.2, the fused colored point cloud reached a steady-state size of approximately 900k points, updated at 30 Hz, while the UE5 stereoscopic view was streamed to 90 frame per second. This 3:1 ratio between the point-cloud update rate and the display refresh rate is consistent with the sensor acquisition rates and does not introduce perceivable stutter, since the Niagara particle system decouples point-cloud updates from frame rendering.

Table 8 shows the performance of V-DART with the hardware described in Section 2.2. The rendering pipeline was also benchmarked at up to 120 Hz; however, this left considerably less headroom relative to the tighter 8.3 ms frame-time budget at 120 Hz (compared to 11.1 ms at 90 Hz), and occasional frame-time spikes caused noticeably less stable frame delivery as the refresh rate approached this upper limit. The system was therefore operated at a fixed 90 Hz refresh rate throughout the experiments, consistent with the stable SteamVR frame time reported above (mean 6.1 ms).

**Figure 9:**
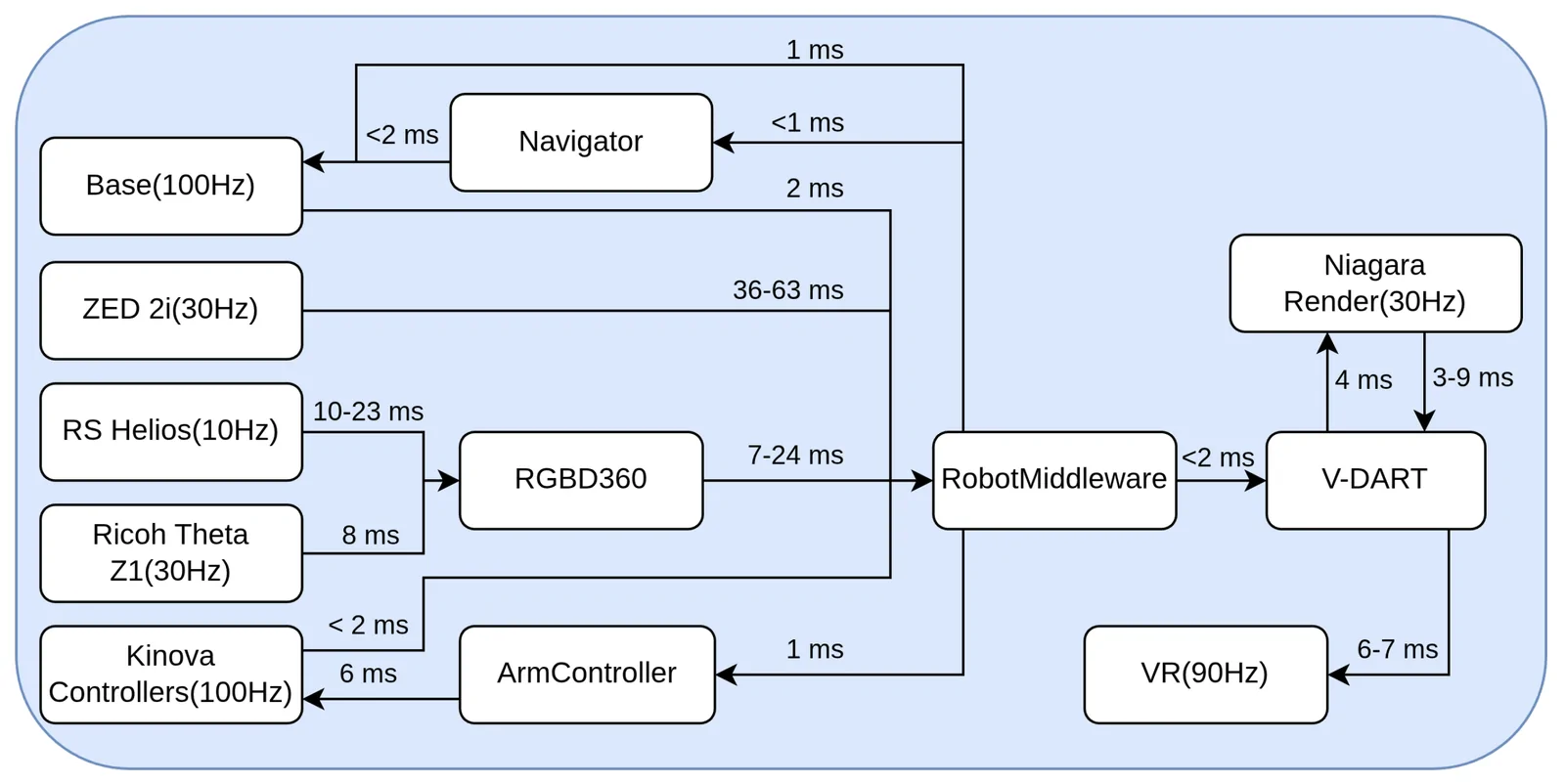
End–to–end latency breakdown of the proposed VR teleoperation system. Adapted from [7] (CC BY 4.0).

Figure 9 presents a block-level breakdown of the system’s end-to-end latency, where each block corresponds to a processing stage, and the connecting arrows denote the associated processing and communication delays across modules.

Among the sensing components, the ZED stereo camera shows the highest overall latency, ranging from 36 to 63 ms. Notably, onboard image processing accounts for only about 7 ms of this total, with the remaining delay attributable mainly to network transmission and synchronization overhead. Given an operating frequency of 30 Hz and a per-frame payload of roughly 56.7 Mibit, available network bandwidth plays a decisive role in shaping the overall latency budget.

For the remaining sensors, the LiDAR unit contributes a latency in the 10–23 ms range, whereas the Ricoh camera adds approximately 8 ms. Both streams are combined in the RGBD360 module, which introduces under 5 ms of additional processing time, together with a synchronization window ranging from 7 to 24 ms, prior to relaying the fused data to the middleware layer.

On the actuation side, the Kinova manipulators achieve a state-transmission latency under 2 ms, with the middleware subsequently requiring around 6 ms for reception and execution at the controller. The navigation module and mobile base follow a comparable pattern, also reporting sub-millisecond transmission delays. With respect to visualization, point cloud data preparation within the Niagara rendering pipeline takes under 14 ms, and the VR rendering step itself completes within a 6–7 ms window per frame.

Taken together, these results indicate that while certain sensing elements—most notably the stereo camera—incur noticeable delays owing to their high bandwidth demands, the rest of the pipeline maintains consistently low and predictable latency values. As a result, the system’s aggregate latency stays within the margins needed for real-time VR teleoperation, supporting responsive user interaction, stable visual feedback, and reliable closed-loop control.

Beyond raw rendering performance, every session is continuously logged for offline use; we now characterize the cost this adds to the pipeline.

### 3.2. Recording Rates, Synchronization, and Logging Cost

Because the logging layer introduced in Section 2.3.5 is a multi-rate, asynchronous set of producers rather than a single synchronous logger, its cadence and computational cost are best characterized per channel. Table 9 reports the recording rate of each record type and the resulting data volume.

All figures reported below were measured with V-DART fully running on the physical P3Bot platform during a normal teleoperation session: the production UE5 application, the RobotMiddleware library and DataRecord were all executing in-process as they do in regular use, with both Kinova arms, the ZED 2i camera and the RoboSense LiDAR streaming live over Ice while the operator moved the robot through the VR interface. The channels driven by operator input (VRPose, VRController, VRHaptic and BaseSpeedCmd) were recorded at their production record size and cadence alongside every channel carrying data received from the robot over Ice.

A session therefore produces 613 records/s and 36.6 KiB/s, i.e., 2.14 MiB per minute, 21.4 MiB for a 10 min demonstration and 129 MiB per hour. Arm joint states account for 38% of the volume, and the four channels driven by the Unreal tick for a further 54%. The two channels whose rate is set by the robot fall slightly below their nominal periods—ArmJoints reaches 186.9 Hz against a 200 Hz loop and RobotPose 66.0 Hz against 66.7 Hz—because each loop only emits a record when the corresponding Ice call has returned. Under position control of the base, BasePoseCmd (41 B) replaces BaseSpeedCmd, raising the total to 38.0 KiB/s. The fused point cloud is excluded from these figures, as it is an optional, disabled-by-default channel discussed separately below.

#### 3.2.1. Acquisition and Transport Times

Table 10 reports the cost of the read-only Ice calls that feed the recorder, measured from the workstation with 100 sequential invocations per service. Two quantities are distinguished. The round trip is measured entirely on the workstation clock and is therefore free of any inter-machine offset; it is dominated by payload size, with the ZED cloud (7.09 MiB per frame, transmitted uncompressed) taking 36 ms against 1.7–5.2 ms for every other service. The sample age is the difference between arrival and the timestamp the producing component wrote, and is the quantity stored in the delay field of every RecordHeader.

#### 3.2.2. Synchronization

As defined in Section 2.3.5 (Table 6), every record is stamped from a single monotonic clock started by startRecording(), so cross-channel alignment reduces to a join on timestamp with a quantum of 1 ms. Measured on the running system, the logger’s own contribution to that timestamp is negligible: 0.9 μs at the median and 31 μs worst-case inside addData(), at least an order of magnitude below the timestamp quantum. Cross-channel alignment is consequently limited by the sampling periods rather than by the logger, and the observed inter-arrival periods track the configured ones closely: 11.0 ms (p99 12.0) for the tick-driven channels, 5.0 ms (p99 11.0) for ArmJoints and 15.0 ms (p99 23.4) for RobotPose. Acquisition latency upstream of the logger is instead recorded in the delay field of every header, so the true acquisition instant can be recovered offline as timestamp−delay; during the campaign this field read 9 ms at the median (p99 15 ms) for ArmJoints, and 100–108 ms (p99 151–173 ms) for the fused cloud. Because every machine on the network is PTP-aligned to sub-millisecond offset (Section 2.4), these figures reflect genuine acquisition and transport delay rather than residual clock skew between the robot and the workstation.

#### 3.2.3. Logging Cost

Table 11 summarizes the cost of the logging layer while the middleware was connected to the robot, with the recorder driven at the session rate of Table 9.

Three properties follow: (i) the recording layer is not a limiting factor for VR responsiveness, with a worst-case frame contribution (31 μs) below 0.3% of the 11.1 ms budget and an aggregate CPU cost that could not be separated from the 10.6% of one core the middleware consumes with the recorder switched off, consistent with the absence of frame drops reported in Section 3.1; (ii) encoding is allocation-free at steady state, since records are appended into pre-reserved fixed-size blocks with no call into the allocator and no reallocation of the accumulated history; and (iii) memory is bounded and predictable, tracking recorded bytes to within 1%, capped at 512 MiB by default (∼4.0 h at the session rate), so that a 30 min demonstration costs only ∼64 MiB, negligible against the 1.6 GB working set reported in Table 8.

#### 3.2.4. Optional Point-Cloud Logging

The fused point cloud is the only variable-length record and is three orders of magnitude more expensive than every other channel combined, so it is treated as a separate, opt-in channel disabled by default. Table 12 reports its cost for the platform’s ∼900k-point-fused cloud, which averaged 880k points over the campaign. Recording at full rate and resolution is not limited by computation but by volume: 151 MiB/s amounts to 89 GiB per 10 min demonstration; decimating to one point in four at 5 Hz brings this down to 8.5 MiB/s, comparable to a compressed video stream while still adding geometry a video cannot provide. Measured against the middleware baseline, the channel costs 3.1% of one core at full rate and resolution and 0.2% in the one-in-four at 5 Hz configuration.

Two effects are visible only on the physical system. First, the nominal 30 Hz is never reached, because each iteration of the fusion loop must first transfer a 7.09 MiB ZED frame (36 ms) and a LiDAR frame (5 ms); the loop delivers 25.1 Hz when one point in four is stored, and storing the whole cloud costs that same thread a further copy that brings it down to 20.0 Hz. Second, and in contrast, the cost of the cloud stays entirely inside the thread that produces it: across every configuration in Table 12, the 99th percentile of the VR thread remains within the run-to-run spread of the reference row, because a cloud record is assembled outside the critical section and only linked into the buffer at the end. Copying the cloud while holding the lock instead raises the VR thread to a 99th percentile of 2.56 ms, a quarter of the frame budget, the distinction that makes the channel usable at all from a thread shared with the VR loop.

Decimation and compression are complementary but do not compose cleanly. Storing the whole cloud and compressing the recorded file offline removes 82% of the bytes, marginally more than the 75% removed by keeping one point in four, and the two together bring a 5 Hz full-resolution stream from 33.5 to 2.5 MiB/s. They do not multiply, however, because decimation erodes what is left to compress: the surviving points are farther apart, their per-channel deltas are larger, and the ratio falls from 5.6× to 3.5× (one point in four) and 2.8× (one in eight), so the combination stores 2.5 MiB/s where a multiplicative model would predict 1.5 MiB/s. Decimation nonetheless remains the only lever that bounds cost at capture time, since it reduces what the recorder writes rather than what a later pass reads. Two caveats apply to these ratios. The best codec tested (per-channel delta followed by xz -6) compresses at 3 MB/s, whereas the fastest configuration, the same delta followed by zstd -1, sustains 541 MB/s on one core but reaches only 3.6×, so a codec cheap enough to run inline would give up part of the ratio. And the robot was stationary throughout the campaign, which makes consecutive clouds and joint vectors more similar than they would be during an actual demonstration; these figures should therefore be read as an upper bound.

### 3.3. Experimental Results

**Figure 10:**
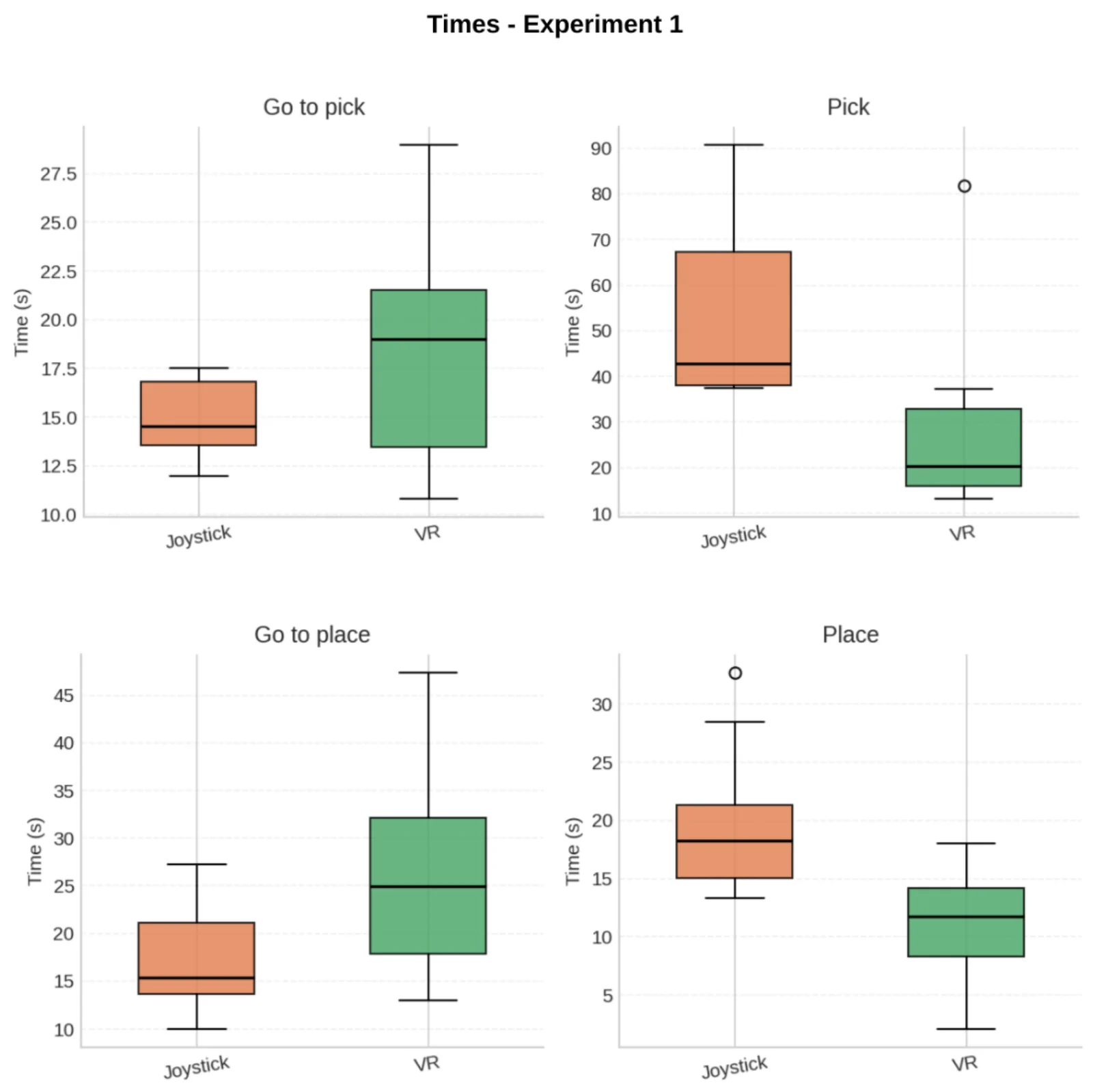
Completion times for Experiment 1, comparing joystick control and VR control (follow mode) on the short-range pick-and-place task. Circles indicate outliers.

**Figure 11:**
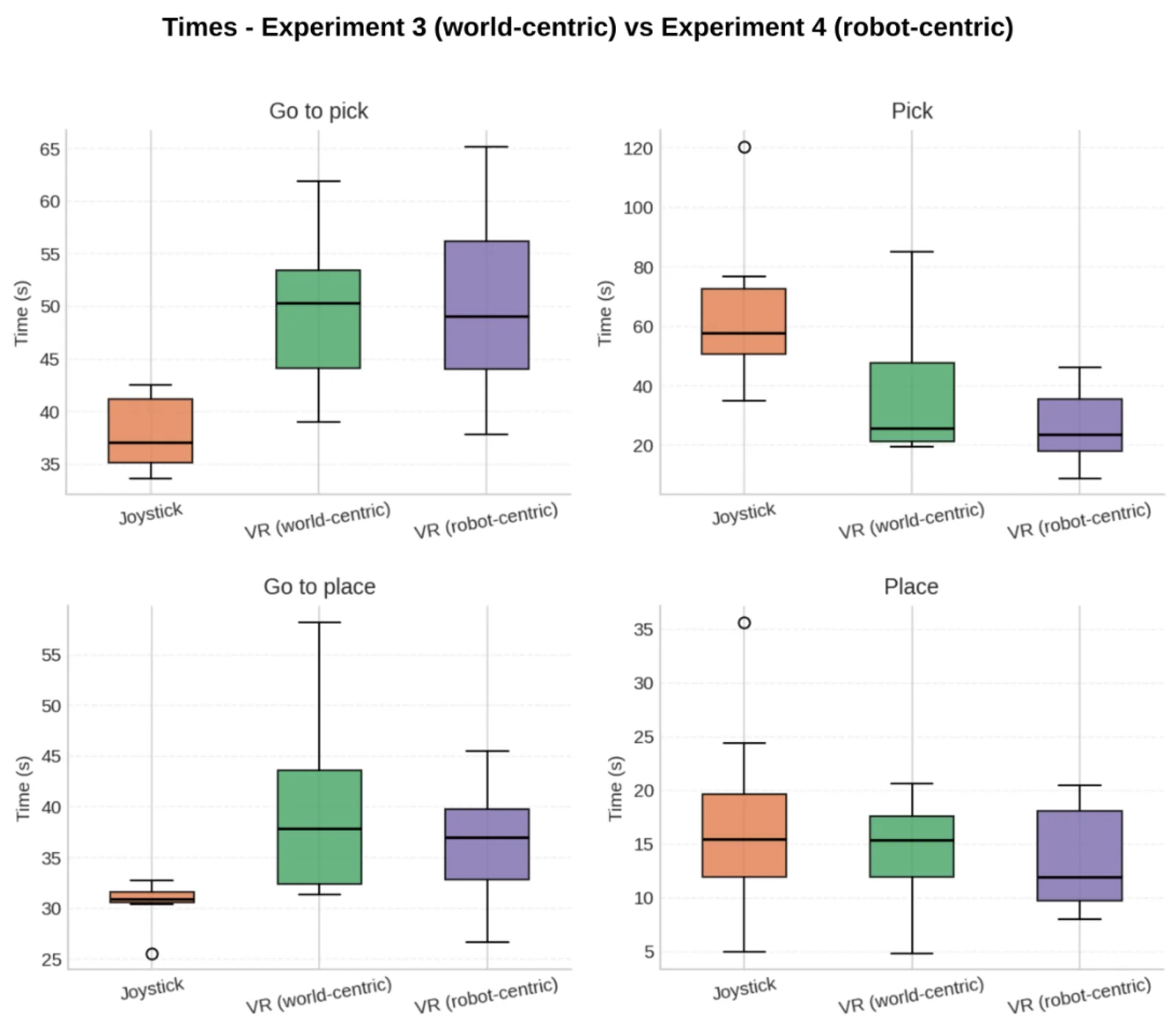
Completion times for Experiments 3 and 4, comparing the world-centric and robot-centric viewpoints on the corridor fetch-and-deliver task. Circles indicate outliers.

**Figure 12:**
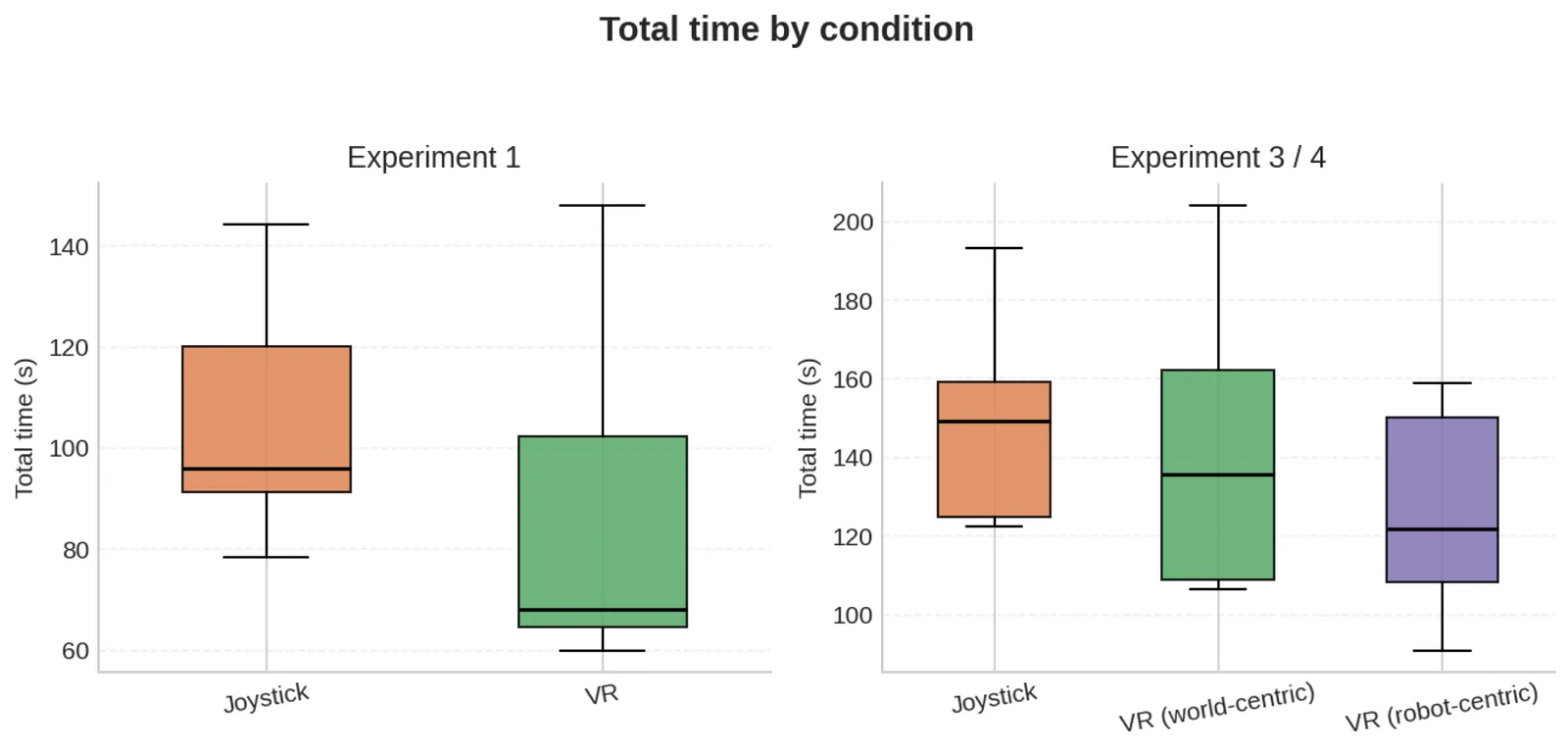
Total completion time by condition for Experiments 1, 3 and 4.

Figure 10, Figure 11 and Figure 12 and Table 13, Table 14 and Table 15 report the completion times, success rates, and collision metrics obtained across the four experiments and control conditions described in Section 2.5.

As shown in Figure 10, joystick control achieved shorter completion times during base navigation (go to pick and go to place phases), whereas VR control yielded better performance in arm manipulation (pick-and-place phases).

Figure 11 shows that joystick control outperforms VR during base navigation, whereas VR achieves shorter completion times in the pick phase; performance during the place phase is comparable between the two conditions.

Figure 12 shows the total completion time for Experiments 1, 3 and 4, with VR control yielding an overall reduction in completion time relative to joystick control.

Table 13 summarizes the mean and standard deviation of pick-and-place completion times for the joystick and VR control conditions in Experiments 1 and 3, together with the corresponding paired Wilcoxon signed-rank test. VR control showed a statistically significant reduction in completion time relative to joystick control during the pick phase in both experiments (p=0.008 and p=0.023, respectively), while the difference during the place phase did not reach significance (p=0.055 and p=0.25).

Table 14 reports the same comparison for the two visualization modes evaluated under VR control (Experiment 3: world-centric; Experiment 4: robot-centric). Robot-centric visualization showed numerically shorter mean completion times in both phases; however, given the small sample size (n = 8) and that neither difference reached statistical significance (*p* = 0.148 for pick, *p* = 0.55 for place), this should be interpreted only as a non-significant trend and not as evidence that robot-centric visualization improves task performance.

Table 15 summarizes task success rates along with the number of object drops and collisions recorded for each experiment and control condition. In Experiment 1, VR control achieved a higher success rate than joystick control (87.5% vs. 75.0%), with fewer object drops (1 vs. 2) and no collisions in either condition. In Experiment 2, joystick control achieved a perfect success rate (100.0%) with no drops or collisions, whereas VR control resulted in a lower success rate (75.0%) and two collisions. In Experiments 3 and 4, both the joystick baseline and the VR conditions (world-centric and robot-centric, respectively) achieved a 100.0% success rate with no object drops or collisions, indicating that performance differences between control and visualization modes in these experiments were reflected primarily in completion time rather than task success.

### 3.4. Subjective Results

**Figure 13:**
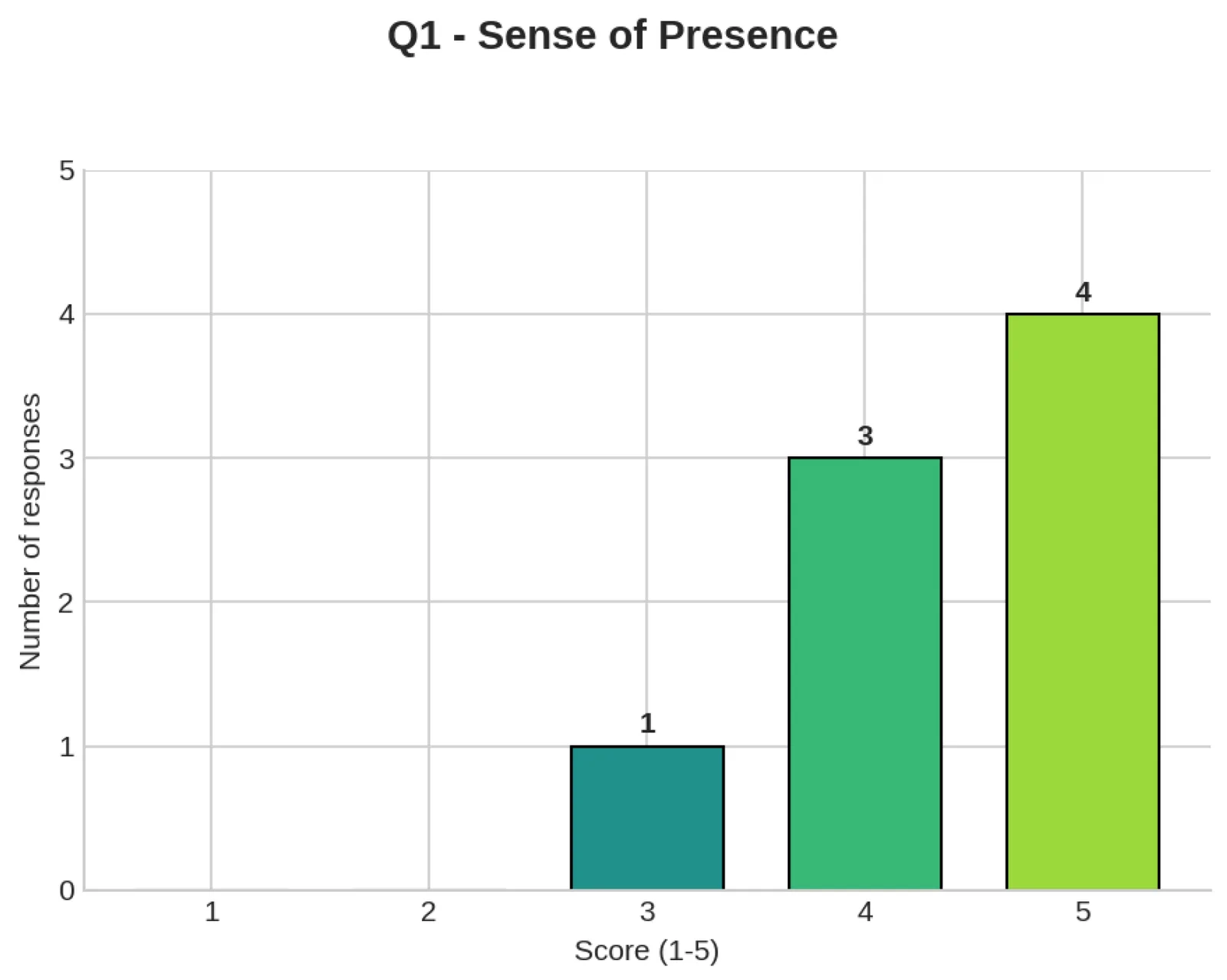
Sense of presence survey results.

**Figure 14:**
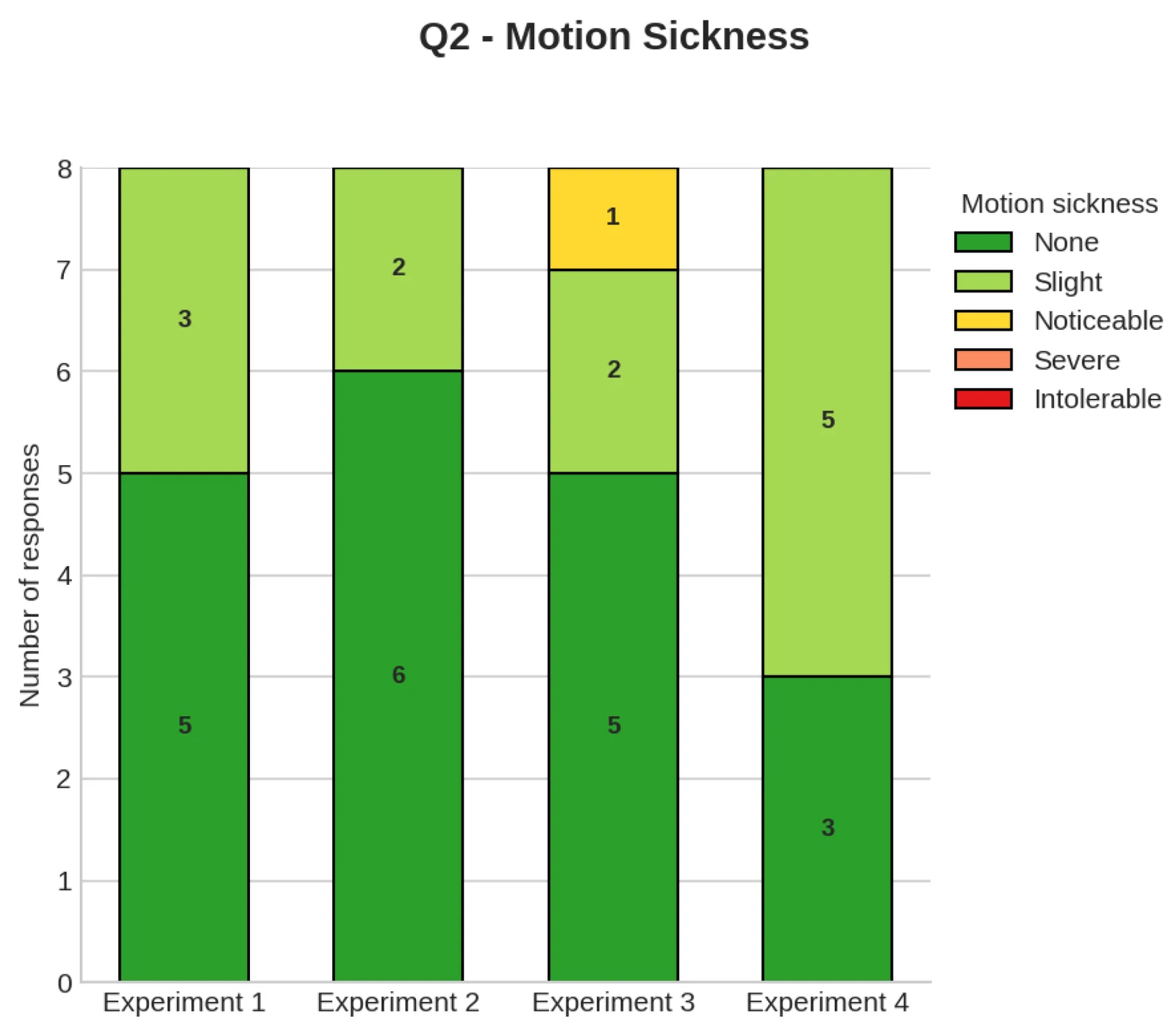
Motion sickness survey results.

**Figure 15:**
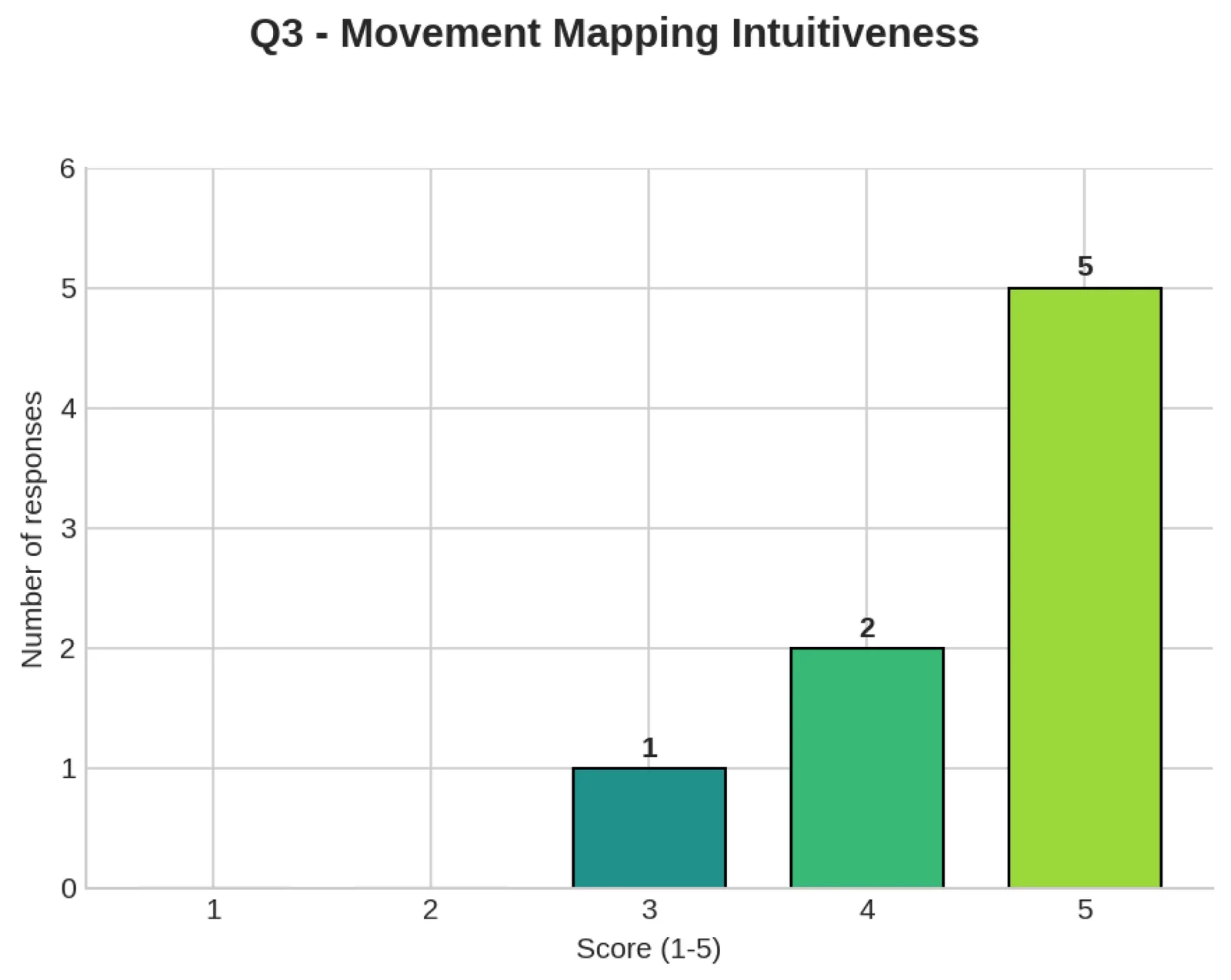
Movement mapping intuitiveness survey results.

**Figure 16:**
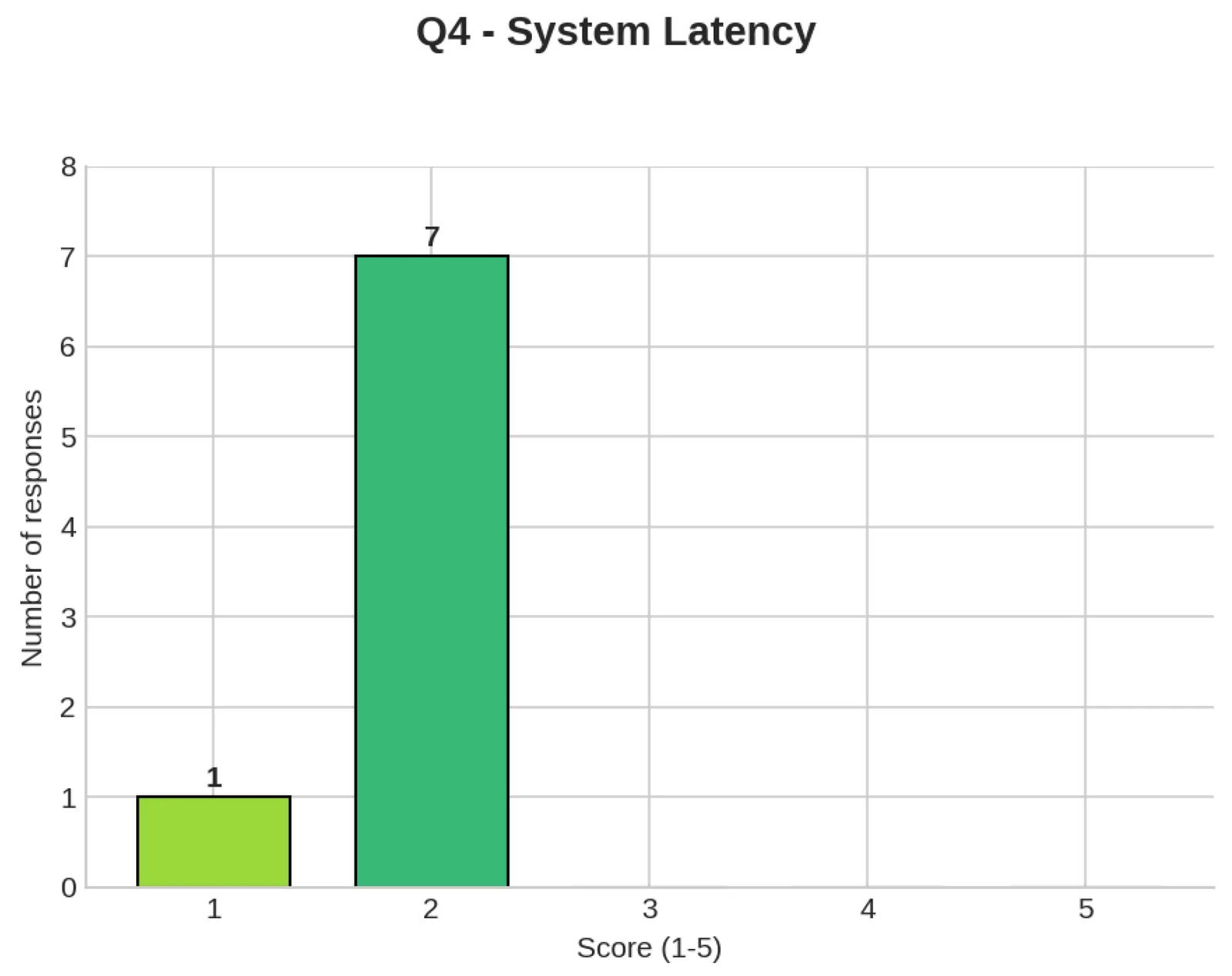
Latency survey results.

**Figure 17:**
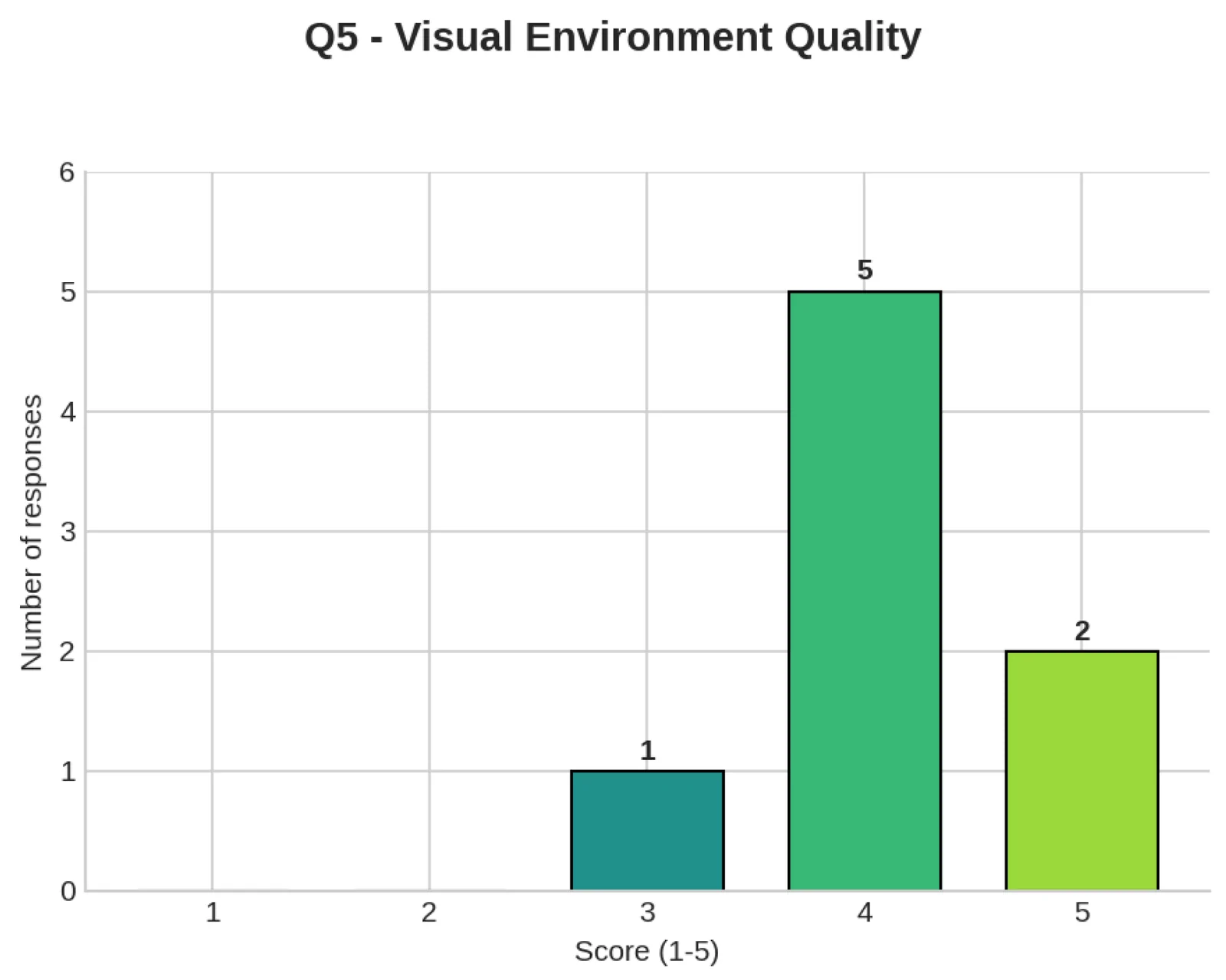
Visual enviroment quality survey results.

1. Did you **feel “present”** in the robot’s environment, as if you were controlling the robot through its “eyes”? 1 (Not Present at All) to 5 (Fully Present)
  As shown in Figure 13, participants reported a high sense of presence, suggesting that the environment visualization effectively conveyed a feeling of embodiment within the robot.
2. Did you experience any symptoms of **motion sickness** (dizziness, nausea, headache) during or after the experiments?—Experiment 1, 2, 3 and 4
  As shown in Figure 14, the majority of participants reported no motion sickness across all experiments except Experiment 4, where a small proportion reported slight discomfort. One participant in Experiment 3 reported noticeable dizziness, attributed to the displacement of the point-cloud visualization during the robot’s turning maneuvers.
3. Were your movements in VR and the movements of the robotic manipulator **intuitive**? 1 (Not Intuitive at All) to 5 (Very Intuitive)
  Figure 15 shows that participants rated the mapping between VR controller movements and robotic arm motion positively, indicating an intuitive control scheme.
4. Did you notice any perceptible **delay** between your actions and the robot’s response/image update? 1 (No Delay) to 5 (Intolerable Delay)
  As shown in Figure 16, participants reported a moderate degree of perceived latency, likely attributable to the robot’s constrained actuation speed rather than to network or rendering delay.
5. Did the virtual environment/robot visualization provide sufficient **detail** to judge distances, sizes, and textures of objects? 1 (Insufficient) to 5 (Very Detailed)
  Figure 17 shows moderate-to-high ratings for visualization detail. Several participants noted that the manipulation-dedicated ZED 2i camera, mounted on the robot’s front, occasionally produced shadows that could hinder object manipulation if not carefully managed.
6. Do you consider V-DART **useful** for tasks in real or hazardous environments? 1 (Useless) to 5 (Very Useful)

**Figure 18:**
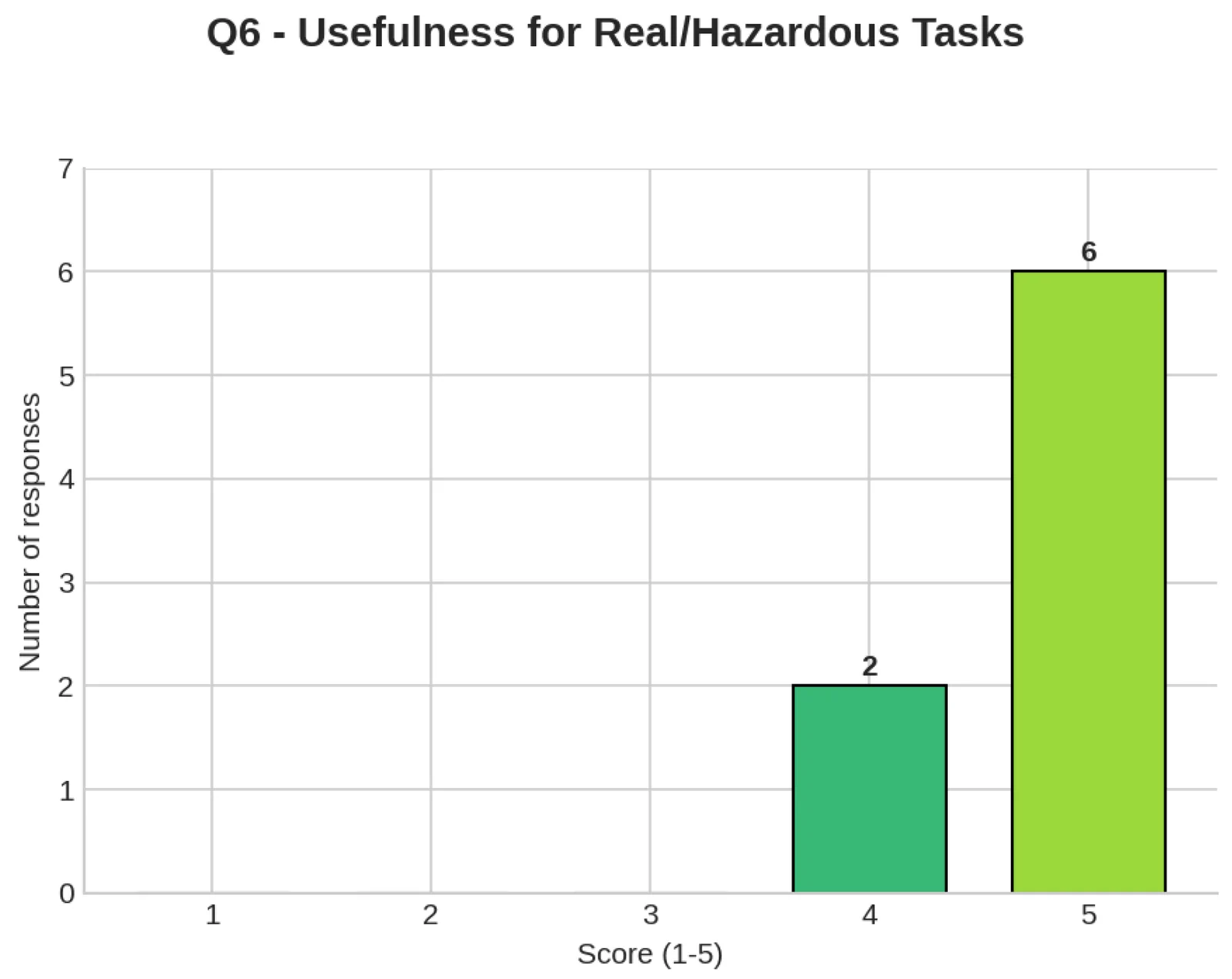
Usefulness for real/hazardous tasks survey results.

As shown in Figure 18, participants considered V-DART useful for real-world or hazardous-environment tasks.

#### Open-Ended Feedback

In addition to the Likert-scale questions above, participants were asked to describe, in open-ended form, what they liked most and what could be improved about the system.

**Positive aspects.** The absence of first-person occlusion afforded by the third-person viewpoint was the most frequently cited advantage (P1, P4, P7, P8), who reported that it improved spatial awareness of the robot within its environment relative to a first-person view. Ease of use and rapid adaptation were also highlighted (P1, P2, P3): despite the number of available controls, participants found the system intuitive after a short practice period. The fidelity and naturalness of the arm motion mapping were valued by P3 and P5, who noted that the robot’s arm replicated their own movements fluidly, including motion captured through the headset’s sensors. A sense of immersion and presence was reported by P4, P6, and P7, who described feeling “inside” the robot and experiencing good synchronization between the virtual and physical environments. Finally, P8 provided detailed positive feedback on the variety of available visualization and control modes, reporting a strong sense of confidence when operating the robot’s arms.

**Difficulties and suggested improvements.** The most frequently reported issue was occlusion of the camera feed and point cloud by the robot’s own arm during manipulation (P1, P3, P4, P8), with participants suggesting the addition of a second, unoccluded viewpoint. Shadows that reduced visibility, particularly when the object was positioned near the center of the robot, were reported by P4 and P6. P2 noted that the robot’s limited locomotion speed, combined with the restricted field of view of the front-mounted ZED 2i camera, constrained situational awareness during base movement. Finally, P8 reported mild initial motion sickness, particularly during turning maneuvers, consistent with the “slight” motion sickness ratings observed in Q2.

## 4. Discussion

### 4.1. Control Mapping: Manipulation Versus Navigation

The comparison between the two control conditions highlights the benefits of the VR control approach for manipulation, while also introducing a different mode of visual feedback that may influence task performance. VR teleoperation significantly outperformed the co-located joystick baseline during the pick phase in both Experiment 1 and Experiment 3 (Table 13, p=0.008, p=0.023). No statistically significant difference between conditions was found during the place phase (p=0.055, p=0.25, respectively); this result should not be interpreted as evidence of an advantage in either direction. This is consistent with the isomorphic, 6-DoF mapping of the VR controllers being better suited to fine manipulation than the non-isomorphic joystick mapping, an advantage largest where positional accuracy matters most (pick) and smaller where the target affords more tolerance (place).

This advantage is further reinforced by the inverse-kinematics solver underlying the VR control loop, which produces fluid arm motion with practically no singularities across the reachable workspace. The traditional joystick scheme, by contrast, frequently required operators to manually adjust intermediate arm configurations to avoid singular postures before reaching the target, adding overhead that is not present in the VR condition. That a remote, latency-affected system outperforms a direct-view baseline suggests the ergonomic benefit of isomorphic mapping outweighs the costs of remote operation. This pattern is corroborated by open-ended feedback, where several participants (P1, P3, P4, P8) identified occlusion of the point cloud by the robot’s own arm as the most frequent difficulty during manipulation, a limitation specific to the remote condition that plausibly contributed to the higher variance observed in VR pick times.

Conversely, the joystick baseline outperformed VR during base navigation across all experiments. Locomotion is a low-dimensional control problem for which a two-axis joystick is already near-optimal, and the operator’s unmediated view likely compensated for any visualization advantage VR might otherwise offer.

This asymmetry is most apparent in the tightest-clearance scenario tested: the only collisions recorded across all experiments occurred in Experiment 2 under VR teleoperation (two collisions, 75% success rate), whereas the co-located joystick condition completed the same doorway-traversal task with a 100% success rate and no collisions. Given the extremely tight clearance involved (approximately 3 cm per side), a 75% success rate under fully remote operation is itself a meaningful result, reflecting reasonably reliable base control even under demanding spatial precision requirements. Nonetheless, the gap relative to the co-located baseline confirms that judging sub-decimeter clearances through a rendered point-cloud visualization remains comparatively more demanding than through direct human vision.

### 4.2. Visualization Mode

Robot-centric visualization (Experiment 4) showed a numerical trend toward shorter completion times over world-centric (Experiment 3) in both phases (6/8 participants); however, given the small sample size (n = 8), this trend did not reach statistical significance and should not be interpreted as evidence that robot-centric visualization outperforms world-centric visualization (Table 14, *p* = 0.148, *p* = 0.55). This quantitative trend contrasts with participants’ subjective preference: most rated world-centric visualization more favorably, consistent with the high presence ratings (Q1) and comments on improved spatial awareness (P1, P4, P7, P8). We consider two non-exclusive explanations for this apparent discrepancy. First, in world-centric visualization, the point cloud is rendered relative to a fixed world frame, so as the robot moves farther from its starting pose it can become small or fall outside the operator’s field of view; periodic rehoming recenters the view on the robot to prevent it from being lost at a distance, and this recentering interrupts the operator’s workflow, plausibly adding overhead not reflected in subjective comfort ratings. Second, since all participants completed Experiment 3 before Experiment 4, part of the time reduction observed under robot-centric visualization may reflect a practice effect from having already performed the fetch-and-deliver task once, rather than the visualization mode alone. Notably, the one reported case of motion sickness occurred under the world-centric condition, attributed to point-cloud displacement during turns. Taken together, these results suggest that the two modes are better suited to different task profiles rather than one being universally preferable: robot-centric visualization may be preferable for short-duration, precision-critical manipulation tasks performed by experienced operators, where the numerical (though non-significant) speed advantage and stable robot-fixed frame outweigh the risk of vection-induced discomfort; whereas world-centric visualization may be preferable for longer sessions, novice operators, or tasks involving extended base navigation, where subjective comfort and situational awareness are prioritized over raw completion time, despite the overhead introduced by periodic recentering.

### 4.3. Latency and Perceptual Quality

Moderate ratings for delay (Q4) and visual detail (Q5) are consistent with the latency breakdown (Figure 9): the ZED stereo camera dominates the budget (36–63 ms), driven mainly by network transmission of its payload rather than onboard processing (7 ms), while all other stages remain in the low-millisecond range, within acceptable bounds for responsive teleoperation despite the wireless link crossing multiple walls. Reported shadowing from the front-mounted ZED 2i (P4, P6) and the arm-occlusion issue point to camera placement and rendering, rather than latency or control mapping, as the main targets for improvement. The root cause is geometric: the ZED 2i is rigidly mounted on the front of the upper body, so whenever an arm moves between the camera and the manipulated object, it necessarily occludes both the RGB-D stream and the resulting point cloud, since the sensor provides only a single, fixed frontal vantage point regardless of the operator’s chosen viewpoint mode. Because this vantage point is physically fixed to the robot’s body, the occluded region cannot be observed directly at that instant, and any recovery of it must rely either on an additional sensor or on previously captured data. Two mitigation strategies follow directly from this diagnosis: (i) adding a second RGB-D camera at a complementary vantage point (e.g., hand-mounted) whose stream could be fused with the frontal view; and (ii) retaining previously captured points in the occluded region as a temporary remanence within the accumulated point cloud, rather than clearing them once the arm blocks the camera’s view. This second, purely software-side approach requires no additional hardware, but cannot reflect genuine changes occurring behind the arm during the occlusion window, so it trades occlusion-filling for a risk of visually stale data in the exact region the operator most needs to see clearly.

### 4.4. System Feasibility

Resource usage (Table 8) remained within sustainable margins on the workstation described in Section 2.2 (Intel Core i9, 14th generation, and an NVIDIA RTX 4080), with GPU utilization (mean 64.29%, max 73.00%) and streaming latency (mean 6.1 ms) leaving sufficient headroom for stable operation without frame drops, consistent with the absence of perceivable stutter and supporting feasibility for sustained, not just short-demonstration, teleoperation sessions.

However, system feasibility was found to be constrained by wireless communication range. Under the two-wall indoor layout used in Experiments 3 and 4, point cloud visualization began to degrade noticeably beyond approximately 30 m from the access point and became unusable for teleoperation beyond 39 m. This degradation is attributed to Wi-Fi signal attenuation through building structures rather than to processing or rendering limitations, and could be mitigated by deploying a mesh network to extend reliable coverage and address this communication bottleneck.

### 4.5. Comparison with Related Work

Table 16 compares V-DART’s rendering performance against IMMERTWIN, one of the closest related digital-twin VR teleoperation frameworks discussed in Table 1 and Table 2. Despite using a comparatively modest GPU (an RTX 4080 versus IMMERTWIN’s RTX 4090), V-DART renders a denser update rate for a smaller point cloud (900k points at 30 Hz, versus 1.6M points at 10 Hz), and sustains a substantially higher rendering rate (90 FPS versus 40 FPS). This is achieved while operating over a wireless link rather than a wired connection, and while reconstructing the environment for a mobile platform rather than a static tabletop setup, a configuration that is inherently more demanding due to the continuously changing sensor viewpoint. Since IMMERTWIN relies on two fixed ZED 2i cameras optimized for a static scene, while V-DART fuses a 360° LiDAR with a single ZED 2i camera on a moving base, the two systems are not directly comparable on a like-for-like task basis; nonetheless, the comparison indicates that V-DART achieves competitive, and in several respects superior, real-time rendering performance under more demanding operating conditions (wireless, mobile) and with less specialized hardware.

### 4.6. Limitations

Key limitations of this study include:

- Small sample size (n=8), which limits statistical power. Given this sample size, all non-significant comparisons reported in this paper (Table 13 and Table 14) should be read strictly as descriptive trends rather than as evidence of a real effect. In particular, the numerically shorter completion times observed under robot-centric visualization do not constitute evidence that this mode outperforms world-centric visualization.
- One participant (P6) showed atypically high pick times in both conditions; their inclusion does not affect the significant results reported.
- Bimanual coordination demand in the joystick condition (two independent controllers) differs from VR’s unified mapping.
- Confounding of control mapping with operating condition (remote vs. co-located): the VR advantage should be read as a lower-bound estimate of the mapping effect alone.
- Operational continuity differs between conditions: in the joystick condition, the operator must switch attention between the base-control and arm-control devices, whereas in VR both arm tracking and base joystick input remain simultaneously available without any such switch. This difference in operational continuity is confounded with both the control-mapping and operating-condition variables discussed above, and may itself contribute to the observed advantage of VR during manipulation. Disentangling all three factors—control mapping, operating condition, and operational continuity—would require a dedicated follow-up study, which we identify as a priority for future work.
- Co-located design of Experiment 1, which limits generalization to other tasks or object geometries.

### 4.7. Future Work

Future work will address the identified limitations, in particular by incorporating a secondary, less occluded viewpoint to mitigate arm self-occlusion, and by performing the first end-to-end validation of the data-logging layer: training and evaluating imitation-learning policies on datasets collected with V-DART. We also plan to extend the evaluation to a larger and more diverse participant pool to increase statistical power for the trends observed here, and to explore additional embodiments under the multi-robot abstraction described in Section 2.3.6.

## 5. Conclusions

We presented V-DART, an extended VR-based teleoperation framework that generalizes our prior stationary dual-arm interface to mobile dual-arm robotic platforms. The system integrates two complementary base-control modalities—joystick input and direct body-tracking—alongside independent, deadman-gated control of both robotic arms, and preserves the bidirectional, real-time communication established in our earlier work. We further introduced a lightweight, self-describing binary logging layer that records asynchronous, multi-rate streams of user pose, controller state, and robot state with negligible runtime overhead, enabling every teleoperation session to double as a structured dataset whose format is designed for compatibility with behaviour cloning and related imitation-learning approaches. The present work implements and characterizes this data-collection layer only; no policy has yet been trained or evaluated on data collected with V-DART.

The latency characterization showed that, despite the added complexity of base locomotion and multi-sensor streaming over Wi-Fi 7, the system maintains an end-to-end latency budget compatible with responsive real-time teleoperation, with the ZED 2i stereo camera constituting the main bottleneck due to network transmission rather than onboard processing. Resource consumption on the operator workstation remained within sustainable margins, supporting extended teleoperation and data-collection sessions rather than only short demonstrations.

The user study, conducted across four tasks with eight participants, indicates that isomorphic VR control of the arms outperforms a co-located joystick baseline during fine manipulation phases, even though the joystick condition benefited from direct, unmediated line of sight, whereas the advantage observed for joystick input during base navigation—particularly in tight-clearance scenarios such as doorway traversal—is more plausibly attributable to the operator’s co-located, direct view of the robot than to the joystick modality itself, since judging sub-decimeter clearances through a rendered point-cloud visualization is inherently more demanding than through direct human vision. Robot-centric visualization showed a non-significant trend toward faster task completion relative to world-centric visualization, while participants subjectively preferred the latter, suggesting that the two viewpoint modes trade off performance against comfort and situational awareness rather than one being strictly superior. Overall, participants reported a high sense of presence, intuitive control mapping, and confidence in the system’s applicability to real or hazardous-environment tasks, with occlusion of the point cloud by the robot’s own arms identified as the most consistent source of difficulty.

These results support the viability of a unified, embodied VR interface for teleoperating mobile dual-arm systems while simultaneously collecting structured expert demonstration data suitable for downstream processing.

## Figures and Tables

**Table 1:** Hardware and interface configuration of V-DART and related teleoperation systems for mobile/bimanual manipulation.

System	Robot	Teleoperation	Display	Sensors
V-DART	Mobile	VR controllers + HMD body tracking	VR HMD (RGB-D twin) ^†^	LiDAR, RGB-D
VR Dual-Arm Teleoperation [7]	Fixed *	VR controllers (arms only)	VR HMD (RGB-D twin) ^†^	LiDAR, RGB-D
IMMERTWIN [16]	Fixed	MR controllers	MR HMD (RGB-D twin) ^§^	RGB-D
Legged MR–VR Teleoperation [5]	Legged	VR/MR gestures	VR/MR HMD (flat video) ^‡^	RGB-D, hand tracking
Embodied Whole-Body [6]	Mobile	VR controllers (WBC/SBC)	VR HMD (flat video) ^‡^/screen	Stereo cameras, mocap
TeleMoMa [9]	Mobile	Vision/VR hybrid	Screen/direct	RGB-D

Abbreviations: HMD, Head-Mounted Display; MR, Mixed Reality; RGB-D, RGB-Depth; SBC/WBC, Single-/Whole-Body Control; VR, Virtual Reality; mocap, Motion Capture. * Mobile chassis; base not teleoperated. † Fused LiDAR + RGB-D point cloud 3D twin; LiDAR adds 360° surround coverage beyond ZED’s frontal FOV. ‡ Flat 2D stereo video in headset; no 3D reconstruction. § RGB-D-only 3D twin (no LiDAR); narrower FOV than V-DART.

**Table 2:** Demonstration data logging capability and experimental results of V-DART and related teleoperation systems.

System	DDL	Main Results
V-DART	Binary, timestamped, multi-rate logger (user pose, controller, robot state)	8 participants; 75–100% success. Up to 44% faster manipulation with VR during the pick phase (*p* < 0.01); high presence and low cybersickness.
VR Dual-Arm Teleoperation [7]	Not reported	17 participants; 76–100% success. High perceived presence and comfort.
IMMERTWIN [16]	Not reported	26 participants; success not quantified. Most participants completed the multi-cube assembly task.
Legged MR–VR Teleoperation [5]	Not reported	10 participants; 95% VR vs. 80% MR success. VR preferred for remote operation; MR performed better on-site.
Embodied Whole-Body [6]	Teleoperation data logged to evaluate downstream IL policy quality	20 participants; ≈94% success. VR increased task time and perceived workload.
TeleMoMa [9]	Demonstrations recorded to train IL policies	18 participants; success not explicitly reported. Hybrid VR+Vision outperformed individual modalities.

Abbreviations: DDL, Demonstration Data Logging; IL, Imitation Learning. Note: “Not reported” indicates the cited work does not describe collecting or logging demonstration data for downstream imitation learning; it does not imply this capability is necessarily absent.

**Table 3:** Hardware components of the P3Bot platform.

Component	Description
Mobile base	Omnidirectional base with passive suspension, providing holonomic motion across uneven indoor surfaces.
Robotic arms	Two Kinova Gen3 7-DoF arms (Kinova Inc., Boisbriand, QC, Canada), each equipped with a Robotiq 2F-85 parallel gripper (Robotiq Inc., Lévis, QC, Canada) as end-effector.
RGB-D camera	Stereolabs ZED 2i stereo RGB-D camera (Stereolabs Inc., San Francisco, CA, USA) mounted on the front of the upper body.
LiDAR/360° camera	RoboSense Helios 3D LiDAR (RoboSense Technology Co., Shenzhen, China) and Ricoh Theta Z1 360° camera (Ricoh Company, Ltd., Tokyo, Japan) mounted at the top of the platform, enabling the construction of colorized point clouds.
Compute unit	ASUS ROG NUC 970 with Intel Core Ultra 9 processor (Intel Corporation, Santa Clara, CA, USA), mobile NVIDIA RTX 4070 GPU (NVIDIA Corporation, Santa Clara, CA, USA), and 32 GB RAM, running Ubuntu 24.04 with the GCC 15 toolchain.
Connectivity	TP-Link BE9300 tri-band Wi-Fi 7 router (TP-Link Technologies Co., Ltd., Shenzhen, China) for high-bandwidth wireless connectivity, plus wired 2.5 GbE Ethernet ports for low-latency local communication.

**Table 4:** Data streams transmitted from P3Bot to the workstation.

Stream	Description
Colored LiDAR point cloud	Produced by the RoboSense LiDAR, with per-point color obtained by projecting the 360° Ricoh Theta Z1 imagery onto the LiDAR returns.
Colored RGB-D point cloud	Produced by the ZED 2i RGB-D camera.
Arm state	Joint angles, applied torques, and motor currents for both arms.
Robot global pose	Pose of the robot in the world frame.

**Table 5:** Control commands transmitted from the workstation to P3Bot.

Command	Description
Gripper commands	Trigger pull maps to gripper closure (1.0: fully closed; 0.0: fully open) for both Robotiq 2F-85 grippers.
Arm set-points	Cartesian set-points for the tool-point of each arm.
Base motion commands	Direct teleoperation mode (instantaneous velocity commands) or navigation-assisted mode (goal poses processed by the on-board navigation stack).

**Table 6:** RecordHeader field layout.

Field	Offset	Type	Description
timestamp	0	uint32_t	Milliseconds since the recording session started (startRecording()); a single monotonic clock shared by every record type, enabling cross-stream synchronization by timestamp.
delay	4	uint32_t	Latency associated with the sample: real acquisition delay when the ICE source provides its own timestamp (e.g., the arm), or polling-loop duration otherwise.
type	8	uint8_t (enum)	Discriminator selecting the payload struct that follows.
payloadSize	9	uint32_t	Payload length in bytes (max. $2^{32}-1$ B, ~4.29 GB per record).

**Table 7:** Record types and their binary payload.

RecordType	Payload	Payload (B)	Record (B)
VRPose	VRData (hmd, left, right Pose)	84	97
VRController	ControllerData (left, right Controller)	62	75
VRHaptic	HapticData (left, right Haptic)	16	29
ColorCloudData	ColorCloudData (X,Y,Z,R,G,B)	variable	—
ArmJoints	JointData (left, right float[8])	64	77
RobotPose	Pose (received base pose)	28	41
BaseSpeedCmd	(x,y,yaw) commanded base velocity	12	25
BasePoseCmd	Pose (commanded base target)	28	41

**Table 8:** Resource consumption of V-DART.

Metric	Mean	Std. Dev.	Maximum
CPU (%)	145.73	23.48	219.80
RAM (MB)	1601.41	20.07	1658.77
GPU (%)	64.29	9.89	73.00
VRAM (MB)	5408.24	668.77	5540.00
SteamVR frame time (ms)	6.1	0.4	6.6

**Table 9:** Per-channel recording cadence and data volume, measured over a 60 s session on the physical platform (90 Hz headset, base under velocity control). The rate of each channel is set by the loop that produces it, not by a common logging clock.

RecordType	Rate (Hz)	Record (B)	Rate (KiB/s)	Share (%)
VRPose	90.0	97	8.53	23.3
VRController	90.0	75	6.59	18.0
BaseSpeedCmd	90.0	25	2.20	6.0
VRHaptic	90.0	29	2.55	7.0
ArmJoints	186.9	77	14.05	38.4
RobotPose	66.0	41	2.64	7.2
**Total**	**612.9**	—	**36.56**	100.0

**Table 10:** Cost of Ice calls supplying the recorder (100 sequential invocations per service). Round trip measured on single clock; sample age measured across machines with PTP sub-millisecond synchronization.

		Round Trip (ms)			Age (ms)
Service	Payload	p50	p99	Max	p50
ZED 2i getColorCloudData	7.09 MiB (826k pts)	36.2	62.5	140.3	46
LiDAR getColorCloudData	494 KiB (57.6k pts)	5.2	9.7	24.1	68
Kinova right getJointsState	8 joints	1.9	6.9	8.6	9
Kinova left getJointsState	8 joints	1.7	4.7	8.4	9
OmniRobot getBaseState	base state	2.0	6.0	8.1	16

**Table 11:** Cost of the data-logging layer at 613 records/s. Per-tick values are medians of five 60 s runs.

Metric	Value
Single addData(), uncontended	31 ns
Single addData(), four concurrent writers	287 ns
Logging time per UE tick (p50/p99/max)	0.9/4.2/31.0 μs
Share of the 11.1 ms frame budget (p99/max)	0.04/0.28 %
Aggregate CPU load	below measurement noise
Heap allocations per record	0
Resident memory/recorded bytes	×1.01
Flush to disk (saveData(), 2.1 MiB)	1 ms

**Table 12:** Cost of the optional point-cloud channel (∼900k-point-fused cloud) with compression ratios (per-channel delta + xz- 6).

Cloud Rate	Points Kept	Achieved	Throughput	Per 10 min	VR Tick p99	Compression
disabled	—	—	36.6 KiB/s	21.4 MiB	4.2 μs	—
30 Hz	all	20.0 Hz	151.0 MiB/s	88.5 GiB	3.8 μs	5.6×
30 Hz	1 in 4	25.1 Hz	47.3 MiB/s	27.7 GiB	4.1 μs	3.5×
5 Hz	all	4.4 Hz	33.5 MiB/s	19.6 GiB	3.8 μs	5.6×
5 Hz	1 in 4	4.5 Hz	8.5 MiB/s	5.0 GiB	4.0 μs	3.5×
1 Hz	1 in 8	1.0 Hz	1.0 MiB/s	587 MiB	4.2 μs	2.8×

**Table 13:** Comparison of joystick and VR control across pick-and-place phases for Experiments 1 and 3.

Experiment	Phase	Joystick (s)	VR (s)	Δ% Faster	*p*
Exp. 1	Pick	53.1±19.9	29.5±22.7	44%	0.008
Exp. 1	Place	19.9±7.0	11.0±5.0	45%	0.055
Exp. 3	Pick	64.6±26.3	37.0±22.9 (world)	43%	0.023
Exp. 3	Place	17.0±9.6	14.5±5.2 (world)	15%	0.25

Note: *p*-values are uncorrected for multiple comparisons (see Section 2.6). Δ% faster values are reported descriptively for all rows, including non-significant comparisons (place phase), and should not be interpreted as evidence of a reliable effect.

**Table 14:** Effect of visualization mode on VR-controlled task completion time (Experiment 3: world-centric vs. Experiment 4: robot-centric).

Phase	VR World-Centric (Exp. 3)	VR Robot-Centric (Exp. 4)	Δ% Faster	*p*
Pick	37.0±22.9	26.3±12.5	29%	0.148
Place	14.5±5.2	13.5±4.9	7%	0.55

**Table 15:** Task success rates and collision metrics.

Experiment	Control	Success Rate (%)	Object Drops	Collisions
Exp. 1	Joystick	75.0	2	0
Exp. 1	VR	87.5	1	0
Exp. 2	Joystick	100.0	0	0
Exp. 2	VR	75.0	0	2
Exp. 3	Joystick	100.0	0	0
Exp. 3	VR (world-centric)	100.0	0	0
Exp. 4	VR (robot-centric)	100.0	0	0

Note: Experiment 4 reports only the VR (robot-centric) condition, isolating the effect of visualization mode using the Experiment 3 joystick trial as the shared baseline reference.

**Table 16:** Performance comparison between IMMERTWIN and V-DART.

Metric	IMMERTWIN [16]	V-DART
GPU Hardware	NVIDIA RTX 4090	NVIDIA RTX 4080
Sensors	2× ZED 2i (fixed)	LiDAR + ZED 2i (mobile)
Point cloud size	1.6M points	900k points
Point cloud update freq.	10 Hz	30 Hz
Deployment	Static setup	Mobile robot
Robot connection	Wired	Wireless (Wi-Fi 7)
Rendering rate	40 FPS	90 FPS

## Data Availability

The source code of V-DART is publicly available at https://github.com/alfiTH/V-DART (accessed on 21 September 2026). The participant data are not publicly available due to the informed consent agreement, which prohibits their dissemination.

## Abbreviations

The following abbreviations are used in this manuscript:

VR	Virtual Reality
UE5	Unreal Engine 5
DoF	Degrees of Freedom
HMD	Head-Mounted Display
PTP	Precision Time Protocol
RGB-D	Red-Green-Blue-Depth (camera)
LiDAR	Light Detection and Ranging
GPU	Graphics Processing Unit
CPU	Central Processing Unit
RAM	Random-Access Memory
VRAM	Video Random-Access Memory
ABI	Application Binary Interface
ICE	Internet Communications Engine (ZeroC middleware)
ROS	Robot Operating System
